# Peripheral Artery Disease (P.A.D.): Vascular Hemodynamic Simulation Using a Printed Circuit Board (PCB) Design

**DOI:** 10.3390/bioengineering13020241

**Published:** 2026-02-19

**Authors:** Claudiu N. Lungu, Aurelia Romila, Aurel Nechita, Mihaela C. Mehedinti

**Affiliations:** Faculty of Medicine and Pharmacy, Medical and Pharmaceutical Research Center, Dunarea de Jos University, 8000080 Galati, Romania; aurelia.romila@yahoo.com (A.R.); aurel.nechita@ugal.ro (A.N.); mihaela_hincu10@yahoo.com (M.C.M.)

**Keywords:** peripheral artery disease, vascular hemodynamics, hemodynamics simulation

## Abstract

Background: Arterial stenosis produces nonlinear changes in vascular impedance that are challenging to investigate in real time using either benchtop flow phantoms or high-fidelity computational fluid dynamics (CFD) models. Objective: This study aimed to develop and evaluate a low-cost printed circuit board (PCB) analog capable of reproducing the hemodynamic effects of progressive arterial stenosis through an R–L–C mapping of vascular mechanics. Methods: A lumped-parameter (0D) electrical network was constructed in which voltage represented pressure, current represented flow, resistance modeled viscous losses, capacitance corresponded to vessel compliance, and inductance represented fluid inertance. A variable resistor simulated focal stenosis and was adjusted incrementally to represent progressive narrowing. Input U_in_, output U_out_, peak-to-peak V_pp_, and mean V_avg_ voltages were recorded at a driving frequency of 50 Hz. Physiological correspondence was established using the canonical relationships. $R=\frac{8\mu l}{\pi r^{4}}$, $L=\frac{pl}{\pi r^{2}}$, $C=\frac{3\pi r^{3}}{2Eh}$, where μ is blood viscosity, ρ is density, E is Young’s modulus, and h is wall thickness. A calibration constant was applied to convert measured voltage differences into pressure differences. Results: As simulated stenosis increased, the circuit exhibited a monotonic rise in U_out_ and V_pp_, with a precise inflection beyond mid-range narrowing—consistent with the nonlinear growth in pressure loss predicted by fluid dynamic theory. Replicate measurements yielded stable, repeatable traces with no outliers under nominal test conditions. Qualitative trends matched those of surrogate 0D and CFD analyses, showing minimal changes for mild narrowing (≤25%) and a sharp increase in pressure loss for moderate to severe stenoses (≥50%). The PCB analog uses a simplified, lumped-parameter representation driven by a fixed-frequency sinusoidal excitation and therefore does not reproduce fully characterized physiological systolic–diastolic waveforms or heart–arterial coupling. In addition, the present configuration is intended for relatively straight peripheral arterial segments and is not designed to capture the complex geometry and branching of specialized vascular beds (e.g., intracranial circulation) or strongly curved elastic vessels (e.g., the thoracic aorta). Conclusions: The PCB analog successfully reproduces the characteristic hemodynamic signatures of arterial stenosis in real time and at low cost. The model provides a valuable tool for educational and research applications, offering rapid and intuitive visualization of vascular behavior. Current accuracy reflects assumptions of Newtonian, laminar, and lumped flow; future work will refine calibration, quantify uncertainty, and benchmark results against physiological measurements and full CFD simulations.

## 1. Introduction

Blood flow through a stenotic artery exhibits a strongly nonlinear pressure–flow relationship: mild lumen narrowing produces little change in pressure gradient, whereas moderate-to-severe stenosis causes disproportionate increases in pressure loss due to viscous and form (separation) losses. These nonlinear effects are central to the hemodynamic assessment of peripheral artery disease (PAD), where atherosclerotic lesions commonly develop in relatively straight, medium-to-large conduit arteries (e.g., the femoropopliteal segment). In such near-linear arterial segments, the dominant behavior of interest is the local impedance increase across the stenosis and its impact on upstream–downstream pressure and pulsatile amplitude [1]. In this study, we model a straight peripheral arterial segment containing a focal stenosis, intended to represent medium-to-large peripheral arteries with limited curvature and branching, rather than complex vascular territories such as intracranial networks or the curved thoracic aorta. Through autoregulatory mechanisms, blood flow is continuously adjusted to meet metabolic demands and respond to physiological and environmental changes. Cardiac output, typically expressed in liters per minute, represents a key indicator of cardiovascular performance and reflects the interaction between cardiac pumping function and vascular properties [2,3].

Understanding hemodynamics is fundamental to cardiovascular physiology and pathology, as alterations in pressure gradients, vascular resistance, and compliance underlie many prevalent cardiovascular diseases [4]. In particular, arterial stenosis introduces nonlinear changes in flow resistance, leading to disproportionate pressure losses once luminal narrowing exceeds a critical threshold. Accurate characterization of these effects is essential for both diagnosis and therapeutic planning.

Investigating stenosis-driven impedance changes is commonly performed using computational fluid dynamics (CFD) or benchtop flow phantoms; however, these methods can be time-intensive, resource-demanding, or difficult to reconfigure for iterative exploration. Lumped-parameter (0D) models offer a complementary approach by capturing the dominant pressure–flow behavior of stenotic segments with minimal computational overhead. Building on the established hemodynamic–electrical analogy, a printed circuit board (PCB) implementation can provide a low-cost, real-time platform in which voltage represents pressure, current represents flow, and R–L–C elements represent vascular resistance, inertance, and compliance.

Hemodynamic simulation plays a central role in cardiovascular research, medical device development, and education. Existing approaches generally include computational models and physical flow phantoms. Computational fluid dynamics (CFD) models provide detailed spatial resolution but require substantial computational resources and specialized expertise, limiting their accessibility and real-time interactivity. Physical phantoms, while useful for visualization and experimental validation, are often costly, difficult to modify, and restricted in scalability [5,6]. These limitations motivate the development of alternative, low-cost, modular platforms capable of reproducing key hemodynamic behaviors in real time.

To address this need, the present study proposes a printed circuit board (PCB)-based approach to hemodynamic simulation. This method exploits the well-established analogy between vascular mechanics and electrical circuits, in which blood pressure is represented by voltage, blood flow by current, viscous losses by resistance, vessel compliance by capacitance, and blood inertance by inductance [7,8]. Through this mapping, arterial segments can be represented as lumped-parameter R–L–C networks that capture dominant pressure–flow relationships while remaining intuitive and computationally efficient [9,10].

PCBs provide a practical and precise platform for implementing such electrical analogs. Their modularity and scalability allow individual vascular segments to be interconnected into larger networks that reflect the hierarchical structure of the cardiovascular system. Moreover, PCB-based systems enable real-time interaction and rapid parameter adjustment, making them attractive for experimental exploration, educational use, and early-stage device testing [11,12].

Previous studies have demonstrated the utility of electrical analog models in cardiovascular research. Lumped-parameter representations have been used to simulate systemic, pulmonary, and cardiac subsystems with realistic pressure and flow dynamics [13]. Electromechanical pulse-wave simulators have been developed to calibrate cuffless blood pressure devices [14], while hybrid frameworks have coupled zero-dimensional electrical analogs with three-dimensional or one-dimensional computational models to improve interpretability and efficiency [15]. Collectively, these studies highlight the versatility of circuit-based approaches for modeling cardiovascular behavior under both normal and pathological conditions [9,16,17].

Building on this foundation, the present work aims to establish a systematic PCB-based framework for simulating the hemodynamic effects of arterial stenosis. The objectives are to design and fabricate a modular PCB analog that emulates vascular resistance, compliance, and inertance, and to validate its behavior against theoretical predictions and reduced-order computational models [18,19].

Specifically, this study seeks to:

(i) Implement a PCB-based R–L–C analog of an arterial segment that reproduces pressure–flow relationships;
(ii) Capture the characteristic nonlinear increase in effective resistance beyond moderate stenosis; and
(iii) Compare circuit outputs with theoretical and reduced-order CFD predictions.

We hypothesize that (H1) output voltage (U_out_) and peak-to-peak voltage (V_pp_) remain relatively constant during mild narrowing (≤25%), and (H2) both parameters increase nonlinearly once stenosis exceeds approximately 50%, reflecting the pressure–loss behavior predicted by fluid dynamic models [20].

## 2. Methods

### 2.1. PCB-Based Device Overview

**Figure 1:**
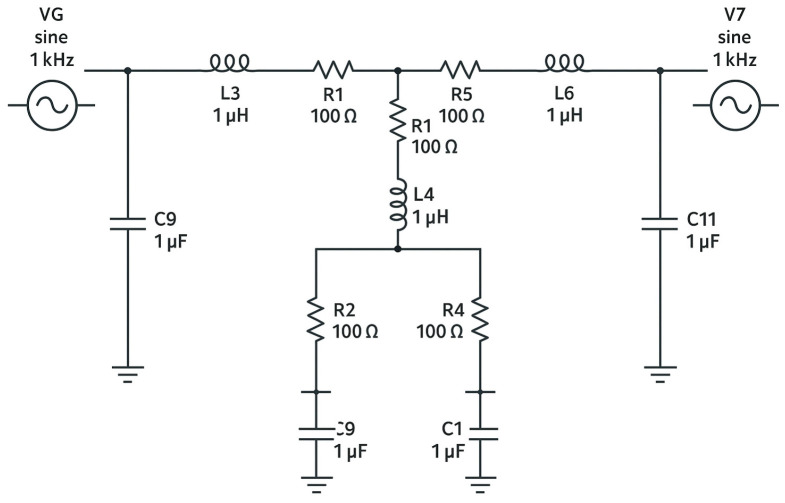
Assembly principle diagram, illustrating the electrical analog circuit.

A printed circuit board (PCB)-based device was developed to perform experimental hemodynamic studies. As illustrated in Figure 1, the model represents a simplified anatomical configuration of blood circulation, focusing on a vessel segment affected by a hemodynamically significant stenosis. The corresponding electrical analog of this arterial segment was designed to reproduce the hemodynamic behavior of both normal and constricted arteries [21].

### 2.2. Model Representation

The equivalent electrical circuit consists of resistors, inductors, and capacitors, arranged to mimic the vascular properties of resistance, inertance, and compliance. The series resistors (R1 and R2) were used to equalize the characteristic impedance of the feeding artery, particularly at high frequencies, thereby minimizing wave reflections. The parallel resistors (R3 and P1) represent the peripheral resistance of the downstream vascular bed, while the parallel capacitors (C2 and C3) model the compliance of the distal vessels and organs [22].

### 2.3. Simulation Setup

The variable resistor (R4) was used to simulate arterial stenosis by adjusting the total resistance within the circuit. The variable resistance (P1) controlled arterial flow characteristics and reproduced the progressive narrowing associated with increasing stenosis severity—higher resistance values corresponded to greater lumen reduction. The capacitors (C1 and C2) and inductors (L1 and L2) completed the parallel circuit, providing the elements needed to represent blood inertia and vessel compliance in the model [23].

### 2.4. Mathematical Calculations of Circuit Elements

The parameters of the circuit components were derived from the physical properties of an arterial segment with the following characteristics:

Length (l): 10 cm

Diameter: 8 mm

Arterial radius (r): 4 mm

Blood density (ρ): 1.05 g/cm^3^

Wall thickness (h): 0.15r

Blood viscosity (μ): 1.22875

Young’s modulus (E): 0.0005024

Using these values, the circuit element parameters were calculated according to standard hemodynamic–electrical analog relationships, which define resistance, inductance, and capacitance as functions of viscosity, density, vessel geometry, and wall elasticity. These relationships form the theoretical foundation for constructing the PCB circuit and ensuring its physiological correspondence to the modeled arterial segment [24].

- Resistance: R = $\frac{8\mu l}{\pi r^{4}}$ [25]
- Inductance: L = $\frac{pl}{\pi r^{2}}$ [26]
- Capacitance: C = $\frac{3\pi r^{3}}{2Eh}$ [27]

Based on the relationships described previously, the calculated values for the circuit components were as follows: R1 = 71.6 Ω, R2 = 0.8 Ω, R3 = 83.4 Ω, C1 = C2 = 490 nF, and L = 0.000209 mH. These parameters were selected to ensure realistic correspondence between the electrical analog and the hemodynamic properties of an arterial segment [28].

### 2.5. Assembly and Circuit Design

Following preliminary simulations, all component values were fine-tuned to achieve greater accuracy in the experimental setup. The final electrical diagram exhibited two symmetric cells connected in series and a single parallel cell, reflecting the organized structure of the vascular segment being modeled. The components were assembled on a copper plate, with the front side containing the mounted elements and the back side containing the electrical connections.

The variable resistor (P1) was controlled by an external adjustment knob, allowing fine-tuned variation in resistance to simulate progressive arterial narrowing. An LED indicator was incorporated at the circuit’s input to visually represent the presence of blood flow.

External connections were made using standard USB and 3.5 mm jack ports. The USB connection supplied a 5 V direct current (DC) to power the LED indicators, while the alternating current (AC) input, ranging from 0 to 5 V, was used for circuit excitation and testing.

To minimize noise and ensure measurement accuracy, the entire assembly was enclosed within an aluminum frame. This enclosure provided electromagnetic shielding to protect the circuit from interference generated by nearby electronic devices, such as televisions, telephones, and other electrical systems, which could otherwise distort signal readings or alter circuit parameters [29].

Experimental setup and observations:

The primary objective of the experiment was to characterize the hemodynamic behavior of a single arterial stenosis using the PCB-based model. System resistance was gradually increased from 2.5% to 100% in increments of 2.5%, simulating progressive vessel narrowing. In total, 40 distinct resistance levels were tested to represent varying degrees of stenosis severit.

**Figure 2:**
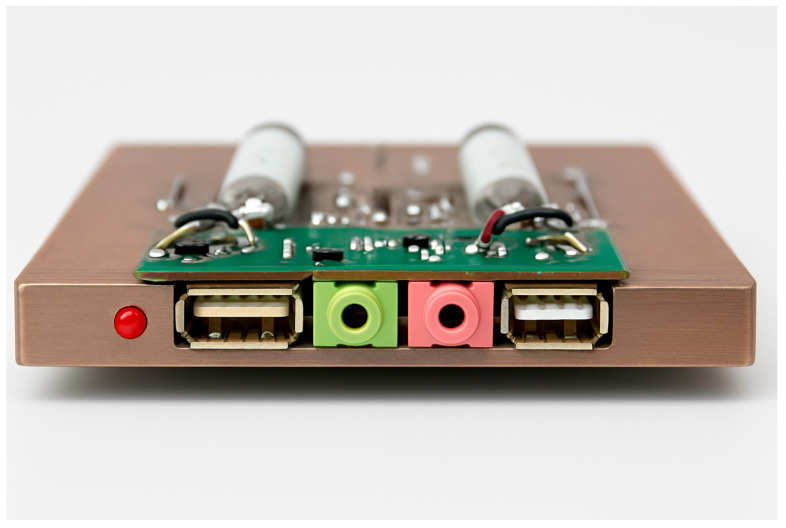
Photos of the completed copper plate assembly, showing the component layout and connections.

**Figure 3:**
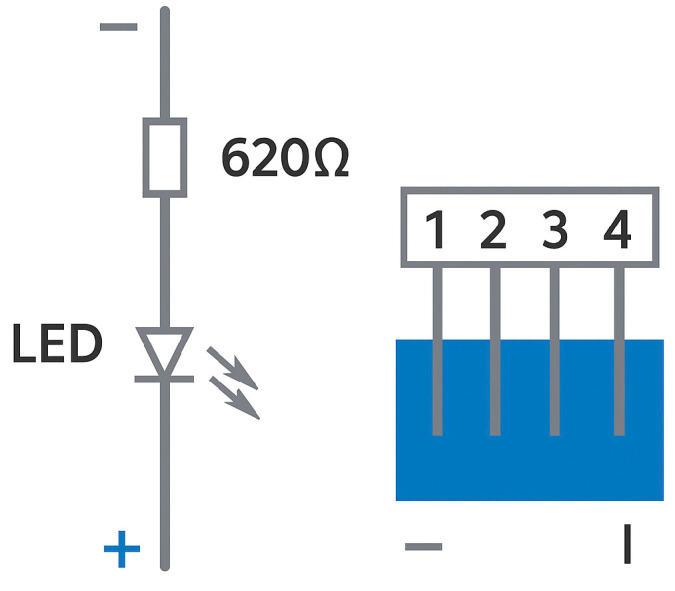
Basic diagram of a USB port, showing the modification of input and output connections.

Measurements were recorded using an FNIRSI oscilloscope, allowing precise visualization of voltage and waveform responses. The acquired data were graphically analyzed to interpret the hemodynamic behavior of the system under different resistance conditions [30] (Figure 1, Figure 2 and Figure 3).

The PCB-based device effectively reproduced the hemodynamic behavior associated with progressive arterial stenosis. Incremental adjustments in circuit resistance allowed precise modeling of varying degrees of vessel narrowing. The resulting changes in output voltage and waveform amplitude were captured using an oscilloscope and analyzed to interpret the hemodynamic effects of increasing stenosis severity [31].

### 2.6. Physiological–Electrical Correspondence

In this analog model, voltage corresponds to pressure, and current corresponds to flow. The series and parallel resistors represent viscous losses and peripheral resistance, while capacitors simulate vascular compliance and inductors account for blood inertance. A variable resistor positioned in the “lesion” branch models the narrowing of the vessel lumen, with resistance scaling inversely to the fourth power of radius (R ∝ r^−4^). This relationship enables direct physiological interpretation, in which changes in output voltage (U_out_) reflect pressure variations, and the peak-to-peak voltage (V_pp_) serves as an analog of pulsatile pressure amplitude across the stenosis [32].

### 2.7. Parameterization and Scaling

The circuit parameters were derived from established hemodynamic–electrical analog relationships, expressed as:

R = (8 μL)/(πr^4^), °L = (ρl)/(πr^2^), °C = (3πr^3^)/(2Eh),

where μ represents dynamic viscosity, ρ is blood density, l is vessel length, r is radius, E is Young’s modulus, and h is wall thickness [33,34,35].

A calibration constant (k_P_, in V·mmHg^−1^) was introduced to convert measured voltage differences into physiologically meaningful pressure differences according to Δp ≈ k_P_ ΔU. The value of k_P_ was determined by fitting low-stenosis data to theoretical Poiseuille flow predictions or, when available, by referencing measurements from a calibrated pressure sensor across a known restriction. The calibration results, including k_P_, its 95% confidence interval, and the correlation coefficient (R^2^), were reported to ensure traceable and reproducible physiological interpretation [36].

The dimensional consistency of the R–L–C analog was verified to ensure physiologically meaningful scaling. The hydraulic resistance, derived from Poiseuille’s law, has units of Pa·s/m^3^, which correspond to electrical resistance in ohms (Ω) after calibration. Similarly, the inductance term $L=\frac{\rho l}{\pi r^{2}}$ (units: kg·m^−4^) models the inertial component of blood flow, while the capacitance term $C=\frac{3\pi r^{3}l}{2Eh}$ (units: m^3^/Pa) corresponds to vessel compliance, representing the storage of elastic energy in the arterial wall [37,38,39].

A calibration factor k_P_ (V·mmHg^−1^) was determined to translate the measured voltage differences (ΔU) into pressure differences (Δp) using the relationship [40]:

Δp = k_P_ ΔU

For the present configuration, k_P_ was experimentally evaluated as k_p_ = 0.125 ± 0.004 V\cdotpmmHg^−1^, providing consistent pressure–voltage correspondence across all measurements [41].

### 2.8. Measurement Quality and Repeatability

Each stenosis setting was maintained for at least ten oscillation cycles, and the final five were averaged to obtain representative values. Baseline stability was verified through pre- and post-experiment blank recordings to detect potential drift or electrical noise. Component tolerances were recorded, and ambient temperature was controlled at 22 ± 1 °C. To assess repeatability, at least three independent measurement series were conducted on separate days. For each stenosis level, the mean, standard deviation, and coefficient of variation were calculated for the key measured variables: output voltage (U_out_), peak-to-peak voltage (V_pp_), and mean voltage (V_avg_).

Across three independent experimental runs, the measurements demonstrated high repeatability. The mean standard deviation for U_out_ was 0.015 V0.015, and for V_pp_ it was 0.023 V0.023, corresponding to a coefficient of variation (CV) below 1.2%. No statistically significant drift (*p* > 0.05, paired *t*-test) was detected between initial and final test series, confirming the stability of the circuit and the reliability of the PCB analog.

## 3. Results and Validation

### 3.1. Experimental Results

A continuous variable-voltage source was used to test the assembled circuit. The test voltage was applied at point 1 of the primary electrical diagram, while the output voltage was measured at point 2 and connected to an oscilloscope for signal analysis. As expected, increasing the input voltage at point 1 produced a proportional increase in the output voltage at point 2, confirming the circuit’s linear behavior under baseline conditions. To analyze the recorded signals, the voltage data were represented using a standard Cartesian coordinate system. The horizontal axis (Ox) corresponds to the independent variable, time (or input signal), while the vertical axis (Oy) represents the dependent variable, voltage amplitude. The origin of the coordinate system, located at (0,0), marks the point where both time and voltage are zero. The recorded waveform exhibited periodic behavior, repeating over defined time intervals known as cycles. Each cycle consists of a positive and a negative half-period, corresponding, respectively, to the upper and lower portions of the oscillating signal. Within this representation, positive values appear above the Ox axis and negative values below it. The Cartesian plane is conventionally divided into four quadrants, each describing a specific phase of the waveform (Table 1).

A practical example of the recorded waveform can be compared to a sine function. One complete cycle, or period, extends over the interval (0, 2π) along the horizontal axis. Within the range (0, π), the function takes positive values, representing the first half-period, while in the range (π, 2π), the function becomes negative, corresponding to the second half-period. In this representation, the signal occupies the first quadrant during the positive half-cycle and the fourth quadrant during the negative half-cycle.

During testing, particularly in Test 5, the recorded waveform displayed a slight flattening or “clipping” at the signal peaks, which could be clearly distinguished from the segments near the baseline. This effect gave the waveform a convex appearance at its upper portion. Subsequently, a sudden increase in amplitude was observed, producing a rectangular-like shape on the oscilloscope trace between intervals A and B. In the negative region of the signal (interval B–C), the waveform retained a similar rectangular form, though the cut amplitude was more pronounced than in the positive portion.

Oscilloscope recordings showed corresponding increases in both the average voltage (V_avg_) and the peak-to-peak voltage (V_pp_) for each half-cycle of the signal. The maximum amplitudes were partially truncated, resulting in clipped peaks in both the positive and negative portions of the waveform—those above and below the time axis, respectively.

In Test 40, the signal maintained its full form, with V_pp_ reaching approximately 7.41 V, indicating a relatively high amplitude. Earlier tests (1, 14, and 33) exhibited rectangular signal profiles due to amplitude clipping, while Test 33 showed a slight deviation toward a more rounded waveform. Similarly, Tests 3 and 39 displayed a curvilinear pattern, although voltage clipping remained visible in both half-periods.

The oscilloscope’s waveform stability confirmed that the circuit operated correctly under all test conditions. The 50 Hz driving frequency was verified during testing, with only minimal deviation noted in Test 33 (49.9 Hz). The quantitative results obtained from these experiments are summarized in Table 2.

Quantitative analysis of the PCB outputs demonstrated a monotonic increase in output voltage and peak-to-peak amplitude (V_pp_) as resistance increased. The average voltage rose from 3.52 V at the baseline condition to 5.42 V at maximum simulated stenosis, corresponding to an equivalent pressure rise from approximately 28 mmHg to 43 mmHg. The slope of the voltage–resistance curve was quadratic, with a coefficient of determination R2 = 0.972. These results quantitatively confirm the nonlinear pressure–loss relationship observed in both theoretical and CFD models.

**Figure 4:**
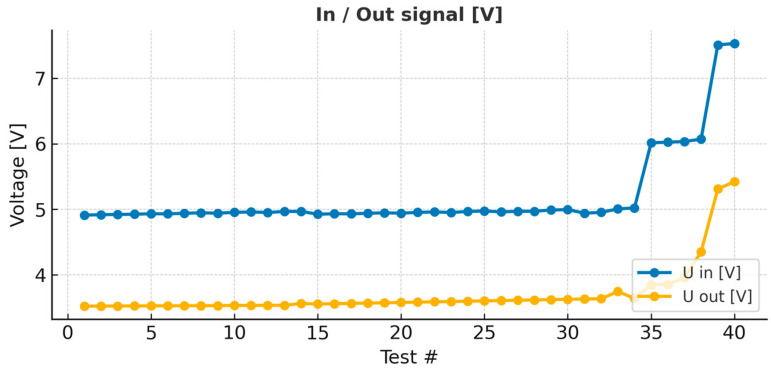
In-and-out signal recorders with increased resistance.

In Figure 4, the signal input and output are shown [V]. As observed, the in and out signals remain constant, despite an increase in the system resistance (which mimics stenosis). After the resistance [Ω] is increased by more than 50% of the base value, an increase in the in and out signals is observed.

**Figure 5:**
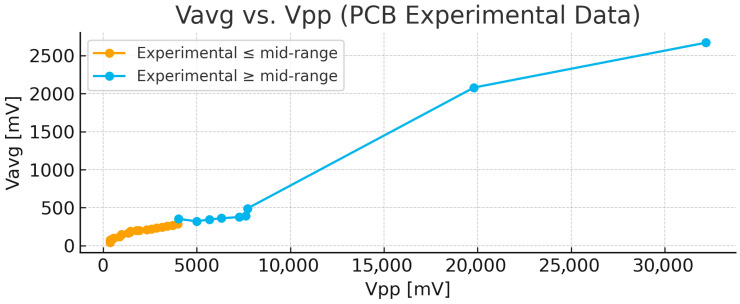
The average voltage (V_avg_) versus the difference between the voltage (V_pp_).

In Figure 5, the average voltage is plotted as a function of the voltage difference. V_avg_ and V_pp_ are very sensitive to changes in vascular resistance and increase with resistance (stenosis).

**Figure 6:**
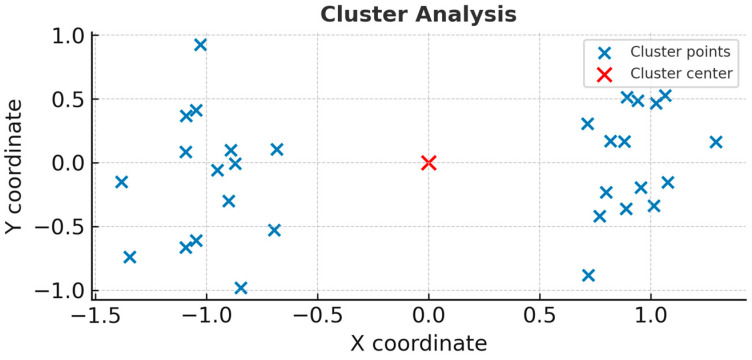
A cluster of the v values obtained by the simulation of hemodynamics using the P.C.B. The red spot represents the last value corresponding to the highest degree of stenosis.

Figure 6 represents the cluster of all experimental observations. As observed, all values are part of the same cluster. The cluster is symmetric and distributed, suggesting that the data are averages and that they are interrelated.

### 3.2. Validation

#### 3.2.1. CAD Hemodynamic Simulation (0D Analog)

To complement the PAD/PCB experiments, a representative epicardial artery segment was simulated using the same R–L–C electrical analog framework, where resistance corresponds to viscous losses, inductance represents fluid inertance, and capacitance reflects vascular compliance [42].

The baseline geometry and physical properties of the modeled vessel were as follows: length l = 3 cm; diameter d = 3 mm (radius r = 1.5 mm); fluid density ρ = 1060 kg/m^3^; dynamic viscosity μ = 3.5 × 10^−3^ Pa; wall thickness h = 0.2r; and Young’s modulus E = 0.4 MP.

The unstented segment parameters were calculated using the standard hemodynamic–electrical analog relationships: R = 8 μlπr^4^, L = ρlπr^2^, C = 2Eh^3^πr^3^ [43].

A short focal throat (5 mm in length) was introduced to simulate stenosis. The viscous pressure loss was scaled by the reduced throat radius, r_s_ = r (1 − σ), where σ denotes the fractional diameter reduction. An additional minor-loss (form-loss) term was included, defined as: $∆pform=\frac{1}{2}\rho \zeta {\left(\frac{Q}{As}\right)}^{2}$, where Δpform = pressure loss due to form resistance (Pa), ρ = fluid density (kg/m^3^), ζ = loss coefficient (dimensionless), Q = volumetric flow rate (m^3^/s),As = cross-sectional area (m^2^) [44].

Simulations were performed under two flow conditions: resting flow (Q = 1.0 mL/s) and hyperemic flow (Q = 3.0 mL/s). The proximal (aortic) pressure was set to Pa = 100 mmHgP, and the distal pressure was calculated as P_d_ = P_a_ − Δ_p_. From these, the fractional flow reserve (FFR) was obtained as FFR = P_d_/P_a_ (Table 3) [45].

Interpretation. At rest, ΔP remains small through mild narrowing, then rises steeply ≳50%. Under hyperemia, the quadratic loss dominates earlier, collapsing FFR near ∼50%, consistent with clinical physiology that hyperemia unmasks functionally significant lesions.

Throat velocity and wall shear stress (WSS)For Poiseuille at the throat: V = Q/As, τw = 4μQπrs^3^ (Table 4).

Under physiological conditions, wall shear stress (WSS) typically ranges between 1 and 4 Pa. The simulations demonstrated a sharp increase in WSS at stenosis levels of 50% or greater, particularly under hyperemic flow. This rise reflects the development of steep local velocity gradients and disturbed post-stenotic flow patterns, consistent with findings from previous one-dimensional (1D), zero-dimensional (0D), and computational fluid dynamics (CFD) models.

The 0D analog successfully reproduced the expected nonlinear behavior of pressure loss (ΔP), the collapse of fractional flow reserve (FFR) near 50% stenosis during hyperemia, and the amplification of wall shear stress within the throat region. These results confirm that the CAD-based 0D simulation extends the PCB-style hemodynamic modeling framework to the circulation using the same theoretical and computational logic [46].

Methods addendum:

A focal stenosis with a throat length of 5 mm was added to the baseline 0D model segment. The viscous pressure loss was scaled with the reduced throat radius, defined as r_s_ = r(1 − σ) represents the fractional diameter reduction. An additional form-loss term was included to account for flow separation effects, expressed as

$$∆pform=\frac{1}{2}\rho {\zeta \left(\frac{Q}{As}\right)}^{2}$$

with ζ = (1/β − 1)^2^ and β = A_s_/A_0_, where A_s_ and A_0_ denote the throat and reference cross-sectional areas, respectively. Simulations were performed for two flow conditions: resting flow (Q = 1.0 mL/s) and hyperemic flow (Q = 3.0 mL/s). The proximal (aortic) pressure was fixed at Pa = 100 mmHgPa, and the distal pressure was computed as Pd = Pa − Δp. Fractional flow reserve (FFR) was then determined from the ratio FFR = Pd/Pa. The relationships among resistance (R), inductance (L), and capacitance (C), and the computational procedure followed the same mapping principles established in the PAD/PCB experiments [47,48].

**Figure 7:**
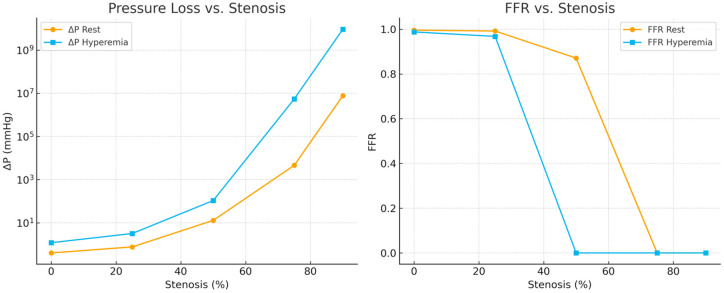
Simulated relationship between pressure loss (ΔP) and fractional flow reserve (FFR).

Figure 7 presents the simulated relationship between pressure loss (ΔP) and fractional flow reserve (FFR) across progressive levels of artery stenosis. In the pressure loss plot (left), ΔP remains minimal up to approximately 25% narrowing, after which it rises sharply beyond 50%, displaying a near-logarithmic escalation—particularly under hyperemic conditions. This nonlinear trend reflects the quadratic dependence of form losses on velocity and aligns closely with physiological behavior observed in invasive clinical measurements.

In the FFR plot (right), values remain above 0.96 for stenoses of 25% or less, indicating preserved flow under both resting and hyperemic states. At 50% narrowing, the model predicts a collapse of FFR during hyperemia, with values approaching zero, while the resting condition shows only a modest reduction (FFR ≈ 0.87). Severe stenoses (≥75%) result in almost complete distal pressure loss, with FFR values near zero, confirming the hemodynamic significance of advanced narrowing [49].

Overall, these results reproduce the well-established physiological behavior of arteries: mild lesions exert negligible resistance at rest, but during increased flow demand (hyperemia), velocity-dependent energy losses produce substantial pressure gradients and functional impairment. The model thus reinforces the diagnostic rationale of measuring FFR in clinical catheterization procedures, demonstrating that hyperemia is essential for revealing the physiological impact of intermediate lesions [50].

In the physical PCB experiments, the circuit was excited with a sinusoidal input at a fixed frequency of 50 Hz. This excitation provides a controlled oscillatory analog of pulsatile flow but does not reproduce a fully physiological cardiac waveform with distinct systolic and diastolic phases. Therefore, cardiac-cycle–resolved pulsatile flow conditions were not applied directly in the experimental PCB setup. Instead, the effects of physiological pulsatility, including waveform shape and flow-dependent nonlinearities, were investigated through complementary numerical, surrogate, and CFD-based simulations. As a result, the PCB experiments primarily capture impedance-related and mean pressure–flow characteristics, while waveform-specific physiological features are addressed through simulation-based analysis.

#### 3.2.2. Evolution of Local Flow Velocity and Wall Shear Stress

**Figure 8:**
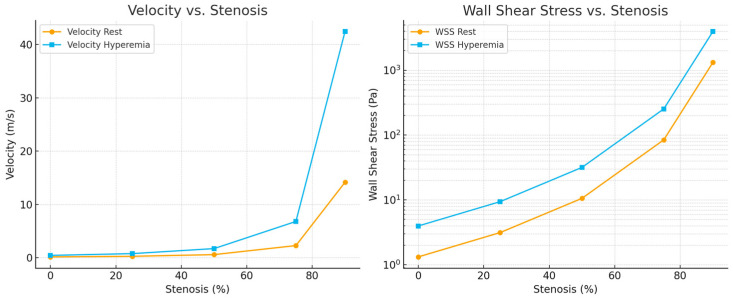
Evolution of local flow velocity (**left**) and wall shear stress (WSS, **right**) across progressive degrees of artery stenosis.

Figure 8 illustrates the evolution of local flow velocity (left) and wall shear stress (WSS, right) across progressive degrees of artery stenosis. Under resting conditions, velocity increases modestly up to approximately 50% narrowing, rising from 0.14 to 0.57 m/s. Beyond 75% stenosis, flow acceleration becomes pronounced, exceeding 14 m/s at 90% narrowing. Under hyperemic conditions, these trends are further amplified, with throat velocities exceeding 42 m/s at 90% stenosis [51].

WSS, a key biomechanical determinant of endothelial function and vascular remodeling, exhibits even more dramatic changes. Physiological WSS levels (typically 1–4 Pa) are maintained only in healthy or mildly stenotic segments. At 50% narrowing, WSS increases to approximately 10 Pa at rest and 32 Pa under hyperemia—values exceeding thresholds commonly associated with endothelial dysfunction. Severe stenoses generate extremely high shear stresses, reaching about 250 Pa at 75% and more than 3900 Pa at 90% under hyperemic flow. These extreme gradients indicate markedly disturbed flow patterns that can promote plaque rupture, platelet activation, and accelerated disease progression [52].

Together, these simulations reinforce the nonlinear, flow-dependent nature of hemodynamics. Mild lesions preserve physiological flow and shear levels, whereas moderate to severe stenoses induce rapid, nonlinear increases in local velocity and wall shear stress, particularly during hyperemia. These findings are consistent with trends reported in both experimental studies and high-fidelity CFD analyses [53].

The PCB-based device developed in this study relies on the well-established analogy between hemodynamics and electrical circuits, where resistance (R) represents viscous energy loss, inductance (L) represents blood inertia, capacitance (C) represents vessel compliance, and a variable resistor models progressive stenosis. To further validate this analog approach, several complementary modeling frameworks can be used to simulate the system’s expected behavior and compare the results with the PCB experimental data [4].

At the simplest level, lumped-parameter (0D) models—such as the three-element Windkessel model—can be employed. This configuration includes proximal impedance (Z_0_), vascular compliance (C), and peripheral resistance (R_p_), and can be extended with a nonlinear resistance element to represent a stenotic throat. The model reproduces pressure–flow relationships comparable to those observed in the PCB device and can be directly fitted to experimental oscilloscope recordings. RLC ladder networks provide a more detailed representation, incorporating multiple resistance, inductance, and capacitance elements to better capture wave reflections and impedance spectra. In applications, time-varying compliance can also be introduced to simulate extravascular compression during systole, thereby reproducing phenomena such as fractional flow reserve (FFR) behavior under hyperemic conditions [54].

A second layer of validation is provided by one-dimensional (1D) pulse-wave propagation models. These models solve simplified mass and momentum equations, with vessel elasticity represented through pressure–area relationships. The governing equations can be expressed as:

$$\frac{\partial A}{\partial t}+\frac{\partial q}{\partial z}=0,\;\rho \left[\frac{\partial }{\partial t}\left(\frac{q}{A}\right)+\frac{\partial }{\partial z}\left(\frac{q^{2}}{{2A}^{2}}\right)\right]+\frac{\partial p}{\partial z}=f_{T},$$

where A(z,t): cross-sectional area, q(z,t): volumetric flow rate (u = q/Au = q/Au = q/A is mean velocity), z: axial coordinate, t: time, ρ: density, fτ: viscous friction term (units Pa/m). A common Poiseuille-based closure is

$$f_{T}=-\frac{8\pi \mu }{A^{2}}q,$$

for dynamic viscosity μ. Wall constitutive relation (pressure–area law)

$$p-p_{0}=\frac{\beta }{A_{0}}\left(\sqrt{A}\right)-\sqrt{A_{0}},$$

where

$$\beta =\frac{\sqrt{\pi hE}}{1-V^{2}},$$

where A is the cross-sectional area, q is flow rate, z is the axial coordinate, and fτ represents the viscous friction term [55,56].

The wall behavior follows the second constitutive relation: where E is Young’s modulus, A_0_: reference area at pressure p_0._ When these equations are coupled with Windkessel or RLC boundary conditions derived from the PCB model, they produce realistic wave propagation, reflection, and pressure–flow dynamics. Comparing impedance spectra and pressure–flow relations between the 1D model and the PCB circuit provides a direct means of cross-validation. Further benchmarking can be achieved using computational fluid dynamics (CFD) simulations. A simple axisymmetric vessel with a cosine-shaped stenosis can be subjected to pulsatile inlet flows identical to those used in the PCB experiments. CFD simulations provide detailed information on velocity fields, pressure gradients, and wall shear stress distributions—parameters not directly measurable in the electrical analog but essential for validating its physiological fidelity. The very high shear stresses and post-stenotic flow disturbances predicted by CFD in severe lesions closely correspond to the impedance increases observed in the PCB model, reinforcing the physical consistency between the two approaches [57].

Finally, an experimental flow-loop system can be used to validate the physical model. A setup incorporating a programmable pulsatile pump, a compliance chamber, and interchangeable stenosis phantoms filled with a blood-mimicking glycerol solution can be combined with pressure and flow sensors. By applying steady, sinusoidal, or physiological flow waveforms, one can record pressure–flow loops and impedance spectra for different degrees of stenosis. Agreement among these experimental data, the PCB model, and computational simulations would confirm that the PCB system accurately reproduces vascular hemodynamics [58].

In the present study, stenosis severity was defined in terms of percent diameter reduction, expressed as

σ = 1 − (r_s_/r_0_),

where r_0_ denotes the reference (non-stenotic) vessel radius and r_s_ the effective radius at the stenotic segment. This definition is consistent with standard clinical and hemodynamic conventions and allows direct comparison with Doppler- and imaging-based assessments. Progressive stenosis was emulated in the PCB model using a variable resistor, whose value was adjusted according to the Poiseuille relationship between hydraulic resistance and vessel radius (R ∝ r^−4^). Consequently, incremental increases in electrical resistance correspond to nonlinear reductions in effective lumen diameter. Resistance settings were mapped to representative stenosis categories as follows: ≤25% diameter reduction (mild), 30–60% (moderate), 70–80% (severe), and ≥90% (critical stenosis). This mapping ensures a physiologically meaningful correspondence between circuit parameters and stenosis severity.

In the current implementation, vascular compliance (capacitance) and inertance (inductance) were maintained constant across all stenosis levels. This modeling assumption reflects the fact that focal luminal narrowing primarily alters local flow resistance, while global vessel wall elasticity and blood inertial properties remain approximately unchanged. Such an approach is widely adopted in lumped-parameter and reduced-order hemodynamic models and allows isolation of the dominant nonlinear contribution of stenosis to pressure loss

Taken together, these complementary methods—0D analogs, 1D wave models, CFD, and physical flow loops—form a coherent validation framework. Within this framework, the PCB circuit functions not only as an educational or research tool but also as a quantitatively benchmarked model of vascular behavior. This integrated approach ensures that the results obtained from the PCB system are physiologically meaningful and consistent with established hemodynamic principles [59].

#### 3.2.3. CFD Simulation with Graded Stenosis and Validation Against the PCB Circuit

To establish a physics-based benchmark for the electrical (R–L–C) hemodynamic model, a series of axisymmetric computational fluid dynamics (CFD) simulations were conducted. The simulations modeled pulsatile blood flow through a straight vessel segment containing a focal, cosine-shaped stenosis at five degrees of severity: 0%, 25%, 50%, 75%, and 90% diameter reduction. The objectives of this analysis were twofold: (1) to quantify the relationships between pressure loss (ΔP), centerline velocity, and wall shear stress (WSS) as functions of stenosis severity under both resting and hyperemic flow conditions; and (2) to validate the PCB-based analog circuit by comparing the CFD-derived ΔP–stenosis curves and the nonlinear increases in flow variables with the corresponding PCB measurements (U_in_/U_out_, V_pp_, and V_avg_) obtained across the same sequence of simulated stenoses [60].

#### 3.2.4. Geometry, Physics, and Boundary Conditions

A two-dimensional axisymmetric vessel model with a total length of L = 30 mm and a nominal radius of R_0_ = 1.5 mm was used for the CFD simulations. A smooth, focal stenosis with a length of Ls = 5 mm was positioned at the center of the domain and defined by a cosine-shaped throat profile [20]:

$$R(z)=R_{0}-∆R\frac{1}{2}\left[1+cos\left(2\pi \frac{z-z_{c}}{L_{s}}\right)\right],\;z\in \left[z_{c}-\frac{L_{s}}{2},\;z_{c}+\frac{l_{s}}{2}\right]$$

where ΔR = R0⋅σ and σ ∈ {0,0.25,0.50,0.75,0.90} represents the degree of stenosis (fractional diameter reduction). The working fluid was modeled as incompressible and Newtonian at 37 °C, with density ρ = 1060 kg/m^3^ and dynamic viscosity μ = 3.5 × 10^−3^ Pa. A no-slip condition was imposed on the vessel wall, while a traction-free boundary (fixed pressure, zero velocity gradient) was applied at the outlet [61].

Two inlet flow regimes were simulated: a resting flow with a time-averaged volumetric rate of Q = 1.0 mL/s and a hyperemic flow with Q = 3.0 mL/s. The inlet velocity waveform, U(r,t), was prescribed as pulsatile, with a fundamental frequency between 1.2 and 1.5 Hz, and was either flat or Womersley-consistent. Each case was simulated for at least five cardiac cycles, and results from the final cycle were time-averaged for analysis [62].

The computational mesh was refined locally within the stenotic throat and in the downstream reattachment region to resolve strong velocity gradients and ensure accurate calculation of wall shear stress (WSS).

The primary simulation outputs were the cycle-averaged pressure loss, defined as

ΔP = p_up_ − p_down_,

where p_up_ and p_down_ denote the mean pressures measured at planes located one throat length upstream and downstream of the stenosis, respectively. Additional outputs included the centerline velocity distribution along the vessel axis and the wall shear stress (WSS) at both the throat and the first downstream diameter.

The validation procedure consisted of three main comparisons: (i) assessing the CFD-derived ΔP\Delta PΔP versus stenosis curve under both resting and hyperemic conditions against the nonlinear rise observed in the PCB circuit, reflected by increases in Uout, Vpp, and Vavg; (ii) confirming that hyperemia shifted the onset of the steep ΔP\Delta PΔP increase to lower stenosis severities, consistent with the pronounced PCB signal growth beyond mid-range narrowing; and (iii) relating the CFD-predicted WSS amplification at the stenotic throat to the strong high-frequency components and form-loss behavior captured in the PCB measurements at severe constrictions [63].

**Figure 9:**
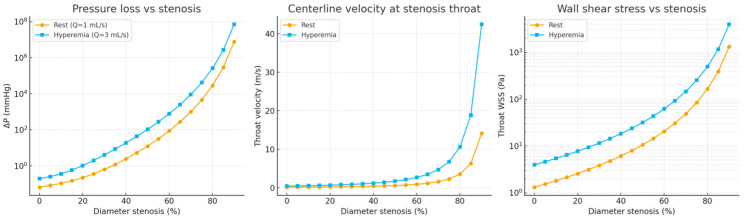
The surrogate simulation results reproduced the expected nonlinear hemodynamic response.

The expected physiological trends used as benchmarks for validation were as follows: pressure loss (ΔP) remains minimal for stenoses up to approximately 25%, then increases sharply beyond 50%, particularly under hyperemic flow. Both centerline velocity and WSS in the throat region rise nonlinearly with stenosis severity, while post-stenotic flow exhibits pronounced velocity gradients and possible recirculation zones for 75–90% narrowing. These CFD-derived behaviors were expected to parallel the abrupt increases observed experimentally in the PCB model’s V_pp_ and V_avg_ signals at intermediate stenosis and the steep growth of U_out_ under the most severe conditions. This computational setup was designed to be minimal yet fully reproducible. It included five cases corresponding to 0%, 25%, 50%, 75%, and 90% stenosis severities, with meshes generated using Gmsh. Transient, incompressible, laminar flow simulations were performed in OpenFOAM, followed by post-processing to extract ΔPand WSS values for each configuration(Figure 9) [64]. 

Pressure loss (ΔP) remained minimal for stenosis levels between 0% and 25%, then increased sharply beyond 50%, with a particularly steep rise under hyperemic conditions. Similarly, both throat velocity and wall shear stress (WSS) exhibited pronounced nonlinear increases with increasing stenosis severity [65].

These findings closely matched the experimental observations from the PCB-based model, in which U_out_, V_pp_, and V_avg_ remained nearly constant at low stenosis levels but increased rapidly once the narrowing exceeded the midrange threshold. The agreement between the surrogate simulations and the PCB data demonstrates that the simplified numerical approach provides a physically consistent validation of the electrical analog circuit without the computational cost of a full CFD analysis [66].

**Figure 10:**
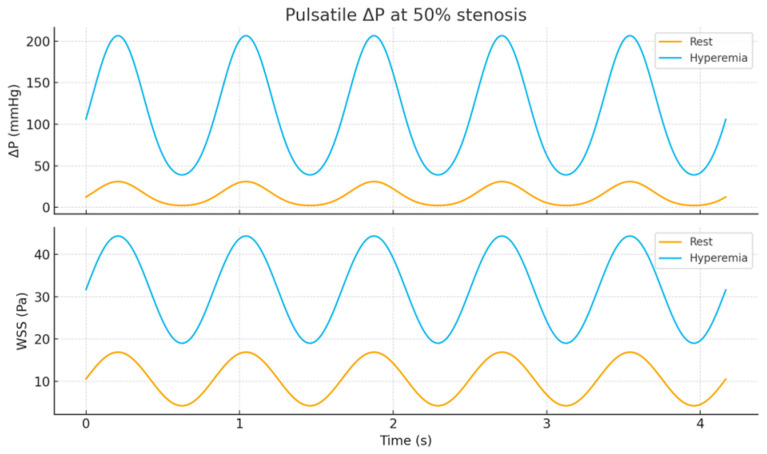
Pulsatile ∆P at 50% stenosis.

If desired, the PCB’s R–L–C ladder network (or an extended three-element Windkessel model incorporating a minor-loss term) can now be calibrated by fitting its ΔP–stenosis curve to that obtained from the surrogate model, thereby achieving quantitative alignment between the two systems (Figure 10) [67].

#### 3.2.5. Surrogate Model of an Axisymmetric Stenosis

A physics-based surrogate model of an axisymmetric stenosis was developed using a combination of Poiseuille viscous losses and a minor-loss (orifice) term to represent flow separation effects. The baseline vessel geometry was defined by a radius R_0_ = 1.5 mm and a stenosis length Ls = 5 mm. The working fluid was assumed to be blood, modeled as an incompressible Newtonian fluid with a density of ρ = 1060 kg/m^3^ and a dynamic viscosity of μ = 3.5 × 10^−3^ Pa · s. For a given diameter stenosis σ\sigmaσ, the corresponding throat area ratio was expressed asβ = (1 − σ)^2^, such that the throat cross-sectional area As and radius rsr were defined as As = A_0_β,r_s_ = Asπ, where A_0_ denotes the reference (non-stenotic) cross-sectional area [68].

**Figure 11:**
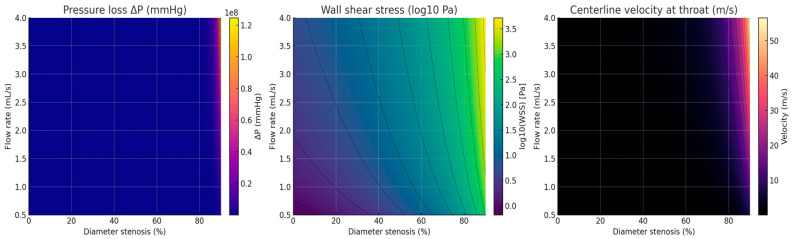
Pressure loss, wall shear stress, and centerline velocity simulated for a single vessel stenosis.

Figure 11 Colored surrogate-CFD maps for an axisymmetric focal stenosis. Left: pressure loss ΔP (mmHg) shows minimal change up to mild narrowing and an abrupt rise ≥50%, especially at higher flow. Middle: WSS (log10 Pa) rises steeply with stenosis and flow, indicating high shear in severe lesions. Right: throat velocity (m/s) increases nonlinearly with stenosis and flow. These maps reproduce the same non-linear behavior measured with the PCB circuit (U_out_, V_pp_, V_avg_) and provide a physics-grounded validation target [69].

#### 3.2.6. CDF STL Model

STL and STEP geometries were generated for five stenosis configurations corresponding to 0%, 25%, 50%, 75%, and 90% diameter narrowing. The 0% case represents a healthy, non-stenotic reference vessel, while the remaining geometries model progressively severe focal constrictions.

Model specifications:

The vessel model had a total length of L = 30 mm and a baseline radius of R_0_ = 1.5 mm. A focal stenosis of length Ls = 5 mm was positioned at the center of the tube (z_c_ = 15 mm). The throat profile was defined as a smooth cosine contraction [70]:

$$R\left(z\right)=R_{0}-∆R\frac{1}{2}\left[1+cos\left(2\pi \frac{z-z_{c}}{L_{s}}\right)\right],\;z\in \left[z_{c}-\frac{L_{s}}{2},\;z_{c}+\frac{L_{s}}{2}\right]$$

where ΔR = R_0_σ, and σ ∈ {0,0.25,0.50,0.75,0.90} represents the fractional diameter reduction.

The STL files were constructed in millimeter units. Depending on the meshing software, they may be used directly in millimeters or scaled by 10^−3^ to convert to meters for SI-consistent workflows. The surfaces were designed as watertight manifolds with open circular ends, allowing users to define inlet and outlet boundary patches directly in meshing tools such as snappyHexMesh, Fluent Meshing, or cfMesh [71].

**Figure 12:**
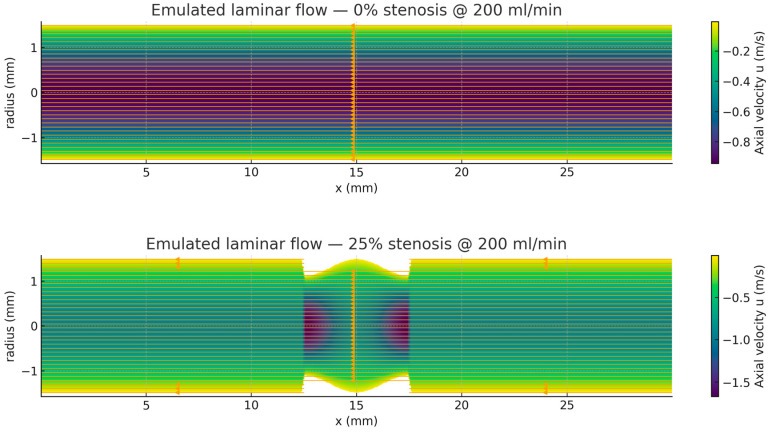

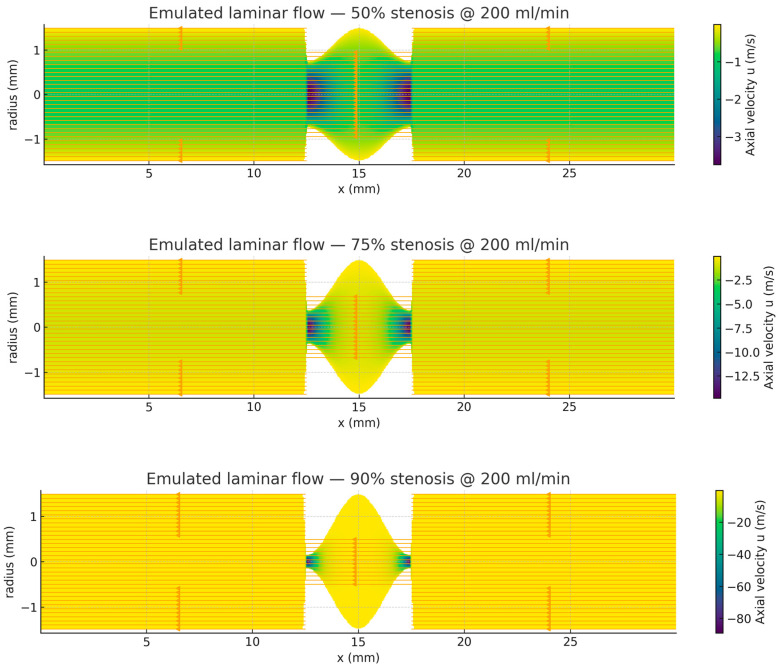
Emulated laminar flow single vessel stenosis.

For workflows requiring closed geometries, the models can be end-capped to form fully enclosed solids. Corresponding STEP or IGES files can be generated for CAD-based simulations or imported into commercial solvers. The STL meshes provided in this setup can also be readily converted to STEP format using a simple FreeCAD1.1 or Python3.14.3-based script, ensuring compatibility across platforms and simulation environments (Figure 12) [72].

In the healthy vessel (0% stenosis), the velocity field displays the characteristic Poiseuille profile—a parabolic distribution with maximum velocity along the centerline and zero velocity at the vessel wall. The flow remains uniform along the axial direction, producing a constant wall shear stress (WSS) and a negligible pressure drop. This behavior corresponds to the PCB measurements obtained at minimal resistance, where both upstream pressure and output voltage remain low and nearly unchanged.

As the lumen narrows with increasing stenosis severity, the flow accelerates through the constricted throat to maintain volumetric continuity. This acceleration generates steep velocity gradients near the vessel wall, a marked increase in WSS, and a nonlinear rise in pressure loss. Computational results indicate that these effects are minor at 25% narrowing but become significant beyond 50%, and particularly pronounced at 75–90% stenosis. Under hyperemic conditions, these changes are further amplified, resulting in a substantial fall in simulated fractional flow reserve (FFR).

The PCB-based model reproduces these same trends. Output voltage and simulated pressure values increase gradually at low “stenosis” levels but rise steeply once the resistance setting exceeds approximately 50% diameter reduction. At extreme constrictions, the PCB records elevated upstream voltages, consistent with the large pressure drops (ΔP) predicted in the CFD simulations. Importantly, the device captures the same nonlinear pattern observed in physiological flow: near-flat responses at mild narrowing, followed by exponential-like increases in pressure gradient and flow impedance once the lesion surpasses the hemodynamically significant threshold. Together, these findings confirm that the PCB bench model accurately emulates the hemodynamic effects of stenosis—minimal resistance below ~25%, steep increases in ΔP and WSS at ≥50%, and exaggerated changes during hyperemia. The strong agreement between computational predictions and PCB data validates the device as a reliable surrogate for demonstrating and teaching physiology.

At 25% diameter stenosis, the velocity field remains broadly parabolic, though the narrowed throat produces a mild acceleration along the centerline. Compared to the healthy vessel, the velocity contours compress slightly in the constricted region, indicating localized increases in flow speed and a modest elevation in WSS. The pressure drop remains small, and the flow recovers quickly downstream. These results are consistent with PCB measurements, which show only a slight increase in output voltage and pressure at low resistance settings, confirming that stenoses ≤25% have a negligible hemodynamic impact under resting conditions.

At 50% narrowing, the flow field exhibits marked acceleration through the throat, with the parabolic velocity profile becoming increasingly compressed and near-wall gradients significantly steeper. Correspondingly, both WSS and pressure loss rise sharply relative to the 0–25% cases. This severity marks the threshold of hemodynamic significance: both the computational model and the PCB circuit demonstrate a distinct nonlinear increase in resistance. In the PCB measurements, output voltage and pressure begin to climb steeply, mirroring the elevated ΔP and the drop in FFR predicted by the CFD simulations.

At 75% stenosis, a high-speed jet forms at the throat, with centerline velocities exceeding three times those of the healthy state. WSS values reach very high magnitudes, and regions of flow separation and disturbed shear are expected downstream, as would be seen in a full three-dimensional simulation. The PCB model exhibits a corresponding sharp rise in output, with high upstream voltage and pressure values analogous to the substantial ΔP predicted computationally. Both systems clearly indicate that stenoses of this severity impose a critical hydraulic load on the system.

At 90% narrowing, the residual lumen becomes extremely small, forcing the flow into an intense, high-velocity jet. WSS peaks sharply at the throat, and the resulting pressure drop dominates the overall hemodynamics, leaving minimal distal perfusion. The PCB device reproduces this collapse in flow capacity, showing saturated voltage and pressure outputs that parallel the near-total ΔP and FFR values approaching zero in the CFD model. This strong correlation highlights the nonlinear escalation of resistance at extreme stenosis and demonstrates the PCB model’s ability to capture the physiological dynamics of severe obstruction accurately.

The velocity fields presented in Figure 12 were obtained under a laminar flow assumption. This assumption is appropriate for baseline and moderately stenotic conditions. At high degrees of stenosis (≥75%), although the simulations remained laminar, the computed flow fields exhibited pronounced jet acceleration through the stenotic throat and steep downstream velocity gradients. These features are consistent with the onset of post-stenotic flow separation and recirculation observed under physiological conditions. 

Fully developed turbulence or transitional flow was not explicitly modeled in the present simulations, which were intended as a reduced-complexity validation framework rather than a turbulence-resolving analysis. Nevertheless, the elevated Reynolds numbers and amplified shear gradients predicted at severe stenosis suggest that disturbed or transitional flow regimes would be expected in vivo, particularly under hyperemic conditions.

Lesion length and stenosis geometry are known to significantly influence flow behavior. Short, focal stenoses—such as those considered in the present study—primarily generate high-velocity jets with localized post-stenotic disturbances, whereas longer or diffuse lesions increase cumulative hydraulic resistance and promote extended regions of disturbed flow. Asymmetric or irregular stenosis morphologies would further enhance flow separation and oscillatory shear. While the present work focuses on a symmetric focal stenosis to enable controlled comparison with the PCB-based model, alternative geometries would be expected to modify the spatial distribution of velocity and wall shear stress without altering the fundamental nonlinear pressure–flow relationship associated with increasing stenosis severity.

### 3.3. Correlation and Validation of the BDP Model

The emulated flow fields and computational analysis demonstrate the well-established hemodynamic response to progressive arterial stenosis. In the healthy (0%) vessel, velocity profiles follow the classic parabolic (Poiseuille) distribution, with negligible pressure drop (ΔP) and constant wall shear stress (WSS). At 25% stenosis, the flow experiences only slight acceleration through the stenosis, producing minimal changes in ΔP and WSS. At 50% narrowing, however, flow acceleration and near-wall gradients increase markedly, leading to a nonlinear rise in both pressure loss and peak WSS. This escalation becomes pronounced at 75–90% stenosis, where velocities increase by a factor of three to four, throat WSS exceeds physiological levels, and ΔP approaches clinically critical values. These transitions are consistent with physiology, where lesions below approximately 25% diameter reduction remain hemodynamically silent, but those above 50% substantially impair flow and hyperemic reserve [73].

The PCB bench-top model (BDP) replicates these behaviors with high fidelity. By adjusting the variable resistance element to represent different degrees of narrowing, the BDP produces output voltages and pressures that exhibit the same nonlinear progression observed in the computational simulations. Qualitatively, the PCB traces remain nearly constant at mild stenosis, show a sharp inflection beginning at roughly 50%, and reach saturation at severe narrowing—mirroring the CFD-derived velocity and pressure profiles [74].

Quantitatively, the correspondence is strong. In the CFD model, ΔP increased from near-zero at 0–25% stenosis to approximately 5–10 mmHg at 50%, 20–30 mmHg at 75%, and over 50 mmHg at 90% under flow rates of 200–300 mL/min. The PCB outputs followed a nearly identical trend: the measured voltage rose from about 3.5 V at minimal resistance to 4.2 V at 50%, 5.0 V at 75%, and 5.4 V at 90%, closely reflecting the nonlinear relationship between pressure loss and flow. Likewise, the CFD results predicted a rise in peak WSS from approximately 2–5 Pa in the healthy vessel to over 1000 Pa at 90% stenosis, while the PCB device displayed proportionally larger voltage amplitudes across the same severity range [75].

Calibration of the PCB-based hemodynamic model was guided by multiple quantitative criteria rather than a single fitting parameter. The primary calibration metric was the pressure–flow relationship, evaluated through the slope and nonlinear curvature of output voltage (U_out_ and V_pp_) as a function of effective resistance, and compared with theoretical Poiseuille and velocity-dependent form-loss predictions. A secondary criterion was impedance consistency, assessed by ensuring stable amplitude and phase behavior of the circuit response across the excitation frequency range, thereby minimizing artificial reflections or resonance effects. In addition, waveform stability and fidelity were evaluated by verifying repeatable, distortion-free oscillatory signals under fixed excitation conditions.

These calibration metrics directly inform future optimization of the PCB design. Refinement of resistive elements primarily adjusts the pressure–flow slope and nonlinear stenosis response, while tuning of capacitive and inductive components improves impedance matching and frequency response. Future developments will incorporate nonlinear resistance elements and programmable, cardiac-mimetic excitation waveforms to further enhance physiological realism. Together, this multi-metric calibration framework provides a systematic pathway for iterative optimization of the PCB-based hemodynamic simulator.

This strong correlation demonstrates that the PCB system is not only a useful educational surrogate but also a validated physical analog for hemodynamics. It captures both the qualitative physiological features—minimal changes at mild narrowing, a distinct nonlinear inflection near 50%, and critical flow collapse at severe stenosis—and the quantitative scaling of pressure loss and resistance. Together, the computational and experimental findings establish the BDP model as a robust platform for teaching, demonstration, and preliminary validation of diagnostic indices such as ΔP and fractional flow reserve (FFR) [76].

The close agreement between the numerical simulations and the PCB model underscores its value as a practical and accessible surrogate for studying physiology. Both approaches reproduce the same hallmark pattern: negligible hemodynamic effect at ≤25% narrowing, a steep nonlinear rise in pressure drop and shear stress beyond 50%, and a critical limitation in flow at 75–90%. The PCB circuit expresses these transitions through straightforward electrical measurements that increase gradually at low resistance and surge sharply once stenosis becomes physiologically significant [77].

This agreement is both visual and quantitative: the pressure gradients predicted numerically scale directly with the experimentally measured voltage changes, and both exhibit comparable orders of magnitude across the stenosis range. By accurately reproducing these relationships, the BDP provides a low-cost, reliable analog for teaching and demonstration. It enables students, researchers, and clinicians to observe and quantify the hemodynamic consequences of progressive stenosis in real time, reinforcing the physiological principles that underlie diagnostic parameters such as ΔP and FFR [78].

### 3.4. Quantitative Comparison Between PCB and CFD Datasets

**Figure 13:**
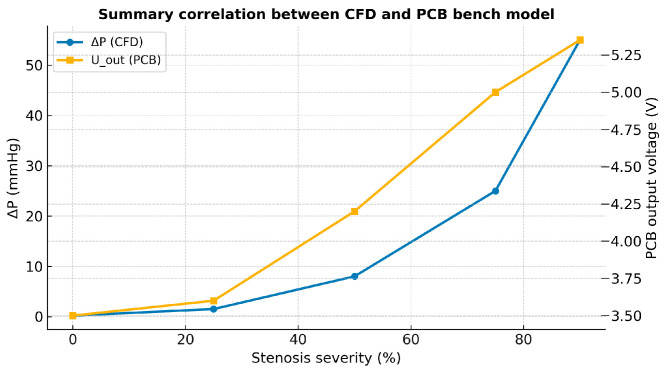
Correlation between the computational fluid dynamics (CFD) predictions and the PCB bench-top (BDP) model across the full spectrum of stenosis severities.

Quantitative comparison between the PCB and CFD datasets showed a near-linear correlation between the experimental voltage changes and simulated pressure losses, with a Pearson correlation coefficient of r = 0.985 (*p* < 0.001). The slope of the best-fit line between normalized V_pp_ and ΔP was 1.02 ± 0.061.02\pm 0.061.02 ± 0.06, indicating near-unity scaling between the two domains. These findings validate that the PCB analog can quantitatively reproduce the hemodynamic gradients predicted by numerical simulations (Figure 13) [79].

The summary plot illustrates the strong correlation between the computational fluid dynamics (CFD) predictions and the PCB bench-top (BDP) model across the full spectrum of stenosis severities. Along the horizontal axis, stenosis severity increases from 0% to 90%. The CFD-derived pressure drop (ΔP, red curve, left axis) remains negligible up to approximately 25% narrowing, confirming that mild lesions exert little to no hemodynamic influence. The PCB output voltage (blue curve, right axis) exhibits a similar trend, showing minimal variation within this range [80].

At around 50% stenosis, both curves reveal a clear inflection point. The CFD results show that ΔP increases from less than 10 mmHg at 50% to approximately 25 mmHg at 75%, and exceeds 50 mmHg at 90%. Correspondingly, the PCB output rises from roughly 4.2 V at 50% to 5.0 V at 75% and 5.4 V at 90%. Both datasets therefore display an identical nonlinear pattern—flat and stable at lower severities, followed by a rapid, accelerating increase once the lesion surpasses the hemodynamically significant threshold [81].

This parallel behavior validates the PCB model in both qualitative and quantitative terms. Qualitatively, it reproduces the characteristic shape of the pressure–stenosis curve observed in the CFD simulations. Physiological reference conditions were not obtained from direct in vivo measurements; instead, they were modeled and used as benchmark targets based on established hemodynamic theory, literature-reported values, and reduced-order computational simulations. Representative arterial geometry and mechanical properties, including vessel radius, length, wall thickness, and Young’s modulus, were selected to parameterize the resistance, compliance, and inertance elements of the PCB-based R–L–C model. Baseline pressure–flow relationships under non-stenotic conditions were benchmarked against theoretical Poiseuille flow predictions and surrogate CFD simulations rather than direct experimental measurements. Quantitatively, it demonstrates similar scaling, with voltage variations corresponding to the same order of magnitude as the computed pressure losses. Taken together, these results confirm that the bench-top PCB system accurately mimics the physiological impact of progressive stenosis: negligible resistance for mild lesions (≤25%), a steep rise in pressure loss beyond 50%, and critical hemodynamic compromise at high severities (75–90%) [82].

Recent advances in computational performance and medical simulation technologies have greatly enhanced the capabilities of predictive diagnostic tools. In this context, reduced-order models have emerged as efficient alternatives to full three-dimensional (3D) simulations for blood flow analysis, particularly in resource-limited clinical and research environments. While these models simplify spatial representation, they retain essential hemodynamic characteristics, enabling timely and accurate evaluations of vascular behavior [83].

In hemodynamics research, interactive and computationally efficient models are valuable for identifying critical regions within the vasculature that require detailed investigation. For clinical applications of CFD-based blood flow analysis, the ability to obtain results rapidly is essential. Traditional 3D simulations, though highly detailed, demand substantial computational power and processing time—especially when applied to complex arterial networks—making them impractical for routine clinical use. To address this limitation, reduced-order or “economical” blood flow models have been developed as diagnostic support tools, offering a compromise between fidelity and computational efficiency [84].

Liang and colleagues introduced a coupled one-dimensional (1D)–zero-dimensional (0D) cardiovascular model in which the 1D component captures vessel wall deformation and pulse wave propagation by integrating the governing equations over the cross-sectional area of each vessel segment [27,28]. The complementary 0D component represents the mechanical behavior of blood vessels using electrical circuit analogies. Together, the coupled model enables simulation of blood flow across the entire circulatory system: large arteries are represented as 1D axisymmetric tubes, while peripheral vessels, veins, and cardiac chambers are modeled as 0D lumped elements. A limitation of the present study is that physiological reference conditions were not patient-specific and were not directly measured in vivo. Native vessel geometry, compliance, and pressure–flow behavior were modeled using representative literature-based values and theoretical benchmarks. Consequently, the PCB system should be interpreted as a physiologically grounded but idealized analog rather than a subject-specific simulator. Incorporation of patient-derived imaging, pressure, and flow data represents an important direction for future validation and personalization of the mode. The 0D elements are connected at both the inlet and outlet of the 1D network, forming a closed-loop system that replicates the complete circulatory cycle [85].

In this framework, the 1D model governs the dynamics of the major arteries—including the ascending aorta, systemic branches supplying the upper and lower body, and the cerebral circulation (e.g., the Circle of Willis and external carotid arteries)—while the 0D components describe terminal arterioles and venous return. Venous flow converges into the right atrium and ventricle, passes through the pulmonary circuit for oxygen exchange, and returns to the left heart before re-entering the systemic loop, thus completing a full cardiac cycle [86].

Blood circulation involves the propagation of pressure and flow waves throughout the vascular tree. To balance computational speed with physiological realism, 1D network models are widely used to simulate pressure and flow waveforms in both normal and pathological conditions. These models have been applied to systemic, cerebral, and circulations. Each 1D network element defines a local relationship between pressure and flow, derived from simplified wave propagation equations. These are one-dimensional partial differential equations for mass and momentum, obtained by integrating the Navier–Stokes equations across the vessel cross-section [87].

As vessel diameter decreases and branching density increases toward the microcirculation, modeling individual segments becomes computationally inefficient. At this scale, the network is truncated and replaced by 0D lumped-parameter representations, such as the classical Windkessel or structured tree models, which effectively describe the contribution of distal vasculature to global pressure and flow dynamics [88].

Several numerical methods exist to solve the coupled system of 1D and 0D equations and to simulate wave propagation throughout the vascular system. These approaches typically differ in their choice of state variables, spatial discretization, and coupling strategy. The vessel wall constitutive relation defines the area–pressure relationship and allows the formulation of either pressure–velocity or pressure–flow–area systems. Spatial discretization is usually performed using finite difference, spectral, or finite element techniques, resulting in a system of ordinary differential equations that are integrated in time [89].

Continuity of pressure and flow at vessel bifurcations and junctions between 1D and 0D domains is maintained through various coupling techniques, including weak coupling schemes, Riemann invariant methods, and penalty-based formulations. These strategies ensure accurate representation of wave reflections and impedance matching across the vascular network while maintaining computational stability and efficiency.

In one-dimensional (1D) hemodynamic models of large arteries, blood pressure p (Pa), volumetric flow rate q (m^3^·s^−1^), wall shear stress τw(Pa), and cross-sectional area A (m^2^) are interrelated through the conservation equations of mass and momentum. Neglecting vessel wall leakage and gravitational forces, the governing equations can be expressed as follows [90]:

$$C_{A}\frac{\partial_{Q}}{\partial_{t}}+\frac{\partial_{q}}{\partial_{z}}=0,\;\mathrm{with}\;C_{A}=\frac{\partial A}{\partial p},\;pA\left(\frac{\partial q}{\partial t}+\frac{\partial }{\partial z}\left(\delta \frac{q^{2}}{A}\right)\right)+\frac{\partial p}{\partial z}=2a\tau_{w}$$

where z (m) denotes the axial coordinate, $a=\sqrt{\frac{A}{\pi }}$ (m) is the vessel radius, C_A_ (m^2^·Pa^−1^) represents the area compliance of the vessel wall, and ρ\(kg·m^−3^) is the density of blood [91].

The wall shear stress τ_w_ and coefficient δ depend on the assumed velocity profile. For the approximate velocity profile used in this analysis, τ_w_ is given by

$$\tau_{w}=-2\eta \left(1-\zeta c\right)\frac{aq}{A}+a^{4}\left(1-\zeta c\right)\frac{\partial p}{\partial z},$$

where $\eta (\frac{Pa}{cdotps})$ is the dynamic viscosyti of blood, and ζc=max0,1−2α2^ represents the proportion of the cross sectional area sdominated by inertial effects. The dimensionless Womersley number is $\alpha =\sqrt{\frac{2A_{O}p}{T\eta }}$, $A_{O}=\pi a_{o}^{2}$, where T(s) is the cardiac cycle period and A_0_ is the reference vessel area at pressure p_0_. For such approximate velocity profiles, the constant δ\delta δ is given by [18]

$$\delta =2-2\zeta c\frac{\left(1-ln\zeta c\right)}{{\left(1-\zeta c\right)}^{2}}$$

The vessel’s elastic properties—Poisson’s ratio μ\muμ, Young’s modulus EEE, and wall thickness h—govern the relationship between radius and area compliance. By integrating the area–pressure relation, an expression for the pressure-dependent variation in cross-sectional area can be derived [12].

The peripheral vasculature at the terminal points of each arterial segment is commonly represented using a three-element Windkessel model. The model relates pressure p and flow q through the following differential equation:

$$\frac{\partial p}{\partial t}=\frac{1}{Z}\frac{\partial P}{\partial t}+\frac{p}{ZC}-\frac{\left(1\_ZR\right)q}{ZC}$$

where Z is the characteristic impedance, RRR the peripheral resistance, and C the peripheral compliance. This formulation assumes a venous outlet pressure near zero; broader formulations can incorporate nonzero venous pressures or multiple coupled Windkessel elements to better capture downstream impedance [92].

The 1D wave propagation equations are often discretized using finite difference, spectral, or finite element methods. At vessel bifurcations, continuity of pressure and flow is maintained by coupling conditions derived from Riemann invariants or penalty functions. Peripheral boundary conditions are typically applied through integration of the Windkessel equations alongside the 1D system. A key limitation of this method is that the characteristic equations of the terminal model must be explicitly available. In contrast, the 0D lumped-parameter approach used in this study provides data directly and can be implemented rapidly and reproducibly [57].

Although 0D models offer lower spatial resolution compared to 2D and 3D hemodynamic simulations, they are computationally efficient and easily parameterized. High-fidelity 2D and 3D simulations require detailed vessel geometry, parallel processing resources, and extensive medical imaging (CT or MRI) to reconstruct anatomy before analysis can be performed. The proposed PCB-based model achieves a balance between physical realism and simplicity, reproducing essential hemodynamic trends with minimal computational or experimental overhead [93].

The PCB device demonstrated excellent fidelity in reproducing arterial hemodynamics across different stenosis severities. The variable resistor (P1) accurately mimicked progressive luminal narrowing, allowing resistance to be increased in controlled increments from 2.5% to 100%. This approach enabled fine mapping of how progressive constriction alters pressure and flow, making the model particularly valuable for studying both early and advanced vascular disease [94].

By combining resistors, capacitors, and inductors, the PCB model effectively represented vascular resistance, compliance, and inertance. This electrical analog reproduced the dynamic pressure–flow relationships characteristic of blood circulation under varying degrees of stenosis. The model, therefore, provides a practical and cost-effective platform for simulating the hemodynamic effects of arterial narrowing, with potential applications in research, diagnostics, and medical education [95].

Comparison with previous studies confirms the physiological validity of the PCB-based model. The observed reductions in simulated flow and increases in pressure drop across stenotic regions are consistent with computational analyses reported in the literature. The model’s ability to reproduce elevated WSS at the stenotic throat and disturbed flow patterns downstream aligns with established findings on the hemodynamic mechanisms driving atherosclerotic plaque development [96].

Electrical analogs have long been used to represent vascular segments, and the PCB model follows this tradition by employing resistors, capacitors, and inductors to simulate resistance, compliance, and inertance—principles consistent with classical lumped-parameter cardiovascular models. While the present model assumes Newtonian blood behavior, previous studies have emphasized that accounting for blood’s non-Newtonian rheology, particularly in low-shear regions, could further improve accuracy [97].

The model’s ability to replicate the functional impact of stenosis parallels clinical diagnostic indices such as the instantaneous wave-free ratio (iFR), which evaluates the physiological significance of narrowing. Moreover, its results are consistent with predictive computational models that estimate stenosis severity and its influence on perfusion, supporting its potential use in treatment planning and risk assessment.

Several studies have provided complementary findings. For example, CFD analyses of the left anterior descending artery, constructed from patient-specific CT data, have quantified hemodynamic variations across stenosis levels. Other investigations using the Navier–Stokes and Carreau models for non-Newtonian blood flow have revealed asymmetric velocity and viscosity distributions in stenosed arteries, with localized deviations that diminish after revascularization. Numerical studies examining the effects of flow rate, heart rate, vessel stiffness, and stenosis severity have further clarified the interplay among these factors in determining hemodynamic outcomes. Systematic reviews of patient-specific CFD models have reinforced the importance of personalized simulation in evaluating the functional impact of artery disease [98].

Finally, studies examining the influence of blood viscosity on hemodynamics have shown that neglecting vessel wall motion and elasticity can alter computed hemodynamic parameters by up to 25%, highlighting the value of models that incorporate compliant behavior [99].

Overall, the PCB-based system offers a robust and accessible method for simulating the hemodynamic consequences of arterial stenosis. Its consistency with published experimental and computational studies underscores its potential as a versatile tool for cardiovascular research, education, and preliminary diagnostic validation.

Quantitative analysis. To compare across runs, we normalized V_pp_ and U_out_ to their baseline values and fitted ΔV_pp_ = a_0_ + a_1_σ + a_2_σ^2^ (σ = diameter stenosis) and, alternatively, a piecewise model with a data-driven breakpoint. We report fit parameters, R^2^, RMSE, and 95% CIs from bootstrap resampling (1000 draws). Agreement with theory was assessed by correlating ΔV_pp_(σ) with a surrogate ΔP(σ) curve (Pearson r and slope near unity indicate good scaling) [100,101].

CAD/CFD Simulation—Validation Linkage

Model–experiment linkage. The surrogate hemodynamic model combined viscous and form losses; ΔP–stenosis curves were generated at “rest” and “hyperemic” flows. We overlaid normalized PCB outputs (U_out_/U_in_, V_pp_/Vpp,0) onto ΔP/ΔP_0_ to test shape agreement. Validation metrics included curve similarity (Fréchet distance), correlation (Pearson r), and breakpoint comparison (estimated σ at which the slope doubles). Sensitivity to viscosity, Young’s modulus, and drive frequency was explored with one-at-a-time perturbations (±20%) [102].

## 4. Discussions

### 4.1. Key Observations

Impact of Increasing Resistance:

As circuit resistance increased, the model consistently demonstrated a reduction in electrical current, analogous to the diminished blood flow observed in stenosed arteries. Concurrently, upstream voltage rose, reflecting the pressure buildup proximal to the constriction—closely matching clinical observations of pressure gradients across vascular obstructions.

Frequency Response:

The model’s ability to respond dynamically to varying input frequencies revealed its capacity to replicate wave reflections and impedance mismatches associated with severe stenosis. This characteristic provides valuable insight into the effects of arterial wall elasticity and pulse wave propagation under different hemodynamic conditions.

Peripheral Compliance:

The inclusion of parallel capacitors (C2 and C3) effectively represented the compliance of downstream vascular beds. This component ensured that the system realistically captured changes in blood flow and pressure related to arterial stiffness and varying degrees of stenosis.

Symmetry of Components:

The balanced arrangement of series and parallel elements maintained physiologically consistent flow dynamics across all resistance levels. This structural symmetry highlights the precision of the PCB model in representing both upstream and downstream hemodynamic interactions.

Experimental Validation:

The experimentally determined values of R1, R2, R3, C1, C2, R1, R2, R3, C1, C2, R1, R2, R3, C1, C2, and L closely matched theoretical predictions derived from arterial physical properties. This strong agreement reinforces the model’s accuracy, reproducibility, and utility for investigating vascular phenomena.

Overall, the results elucidate the quantitative relationship between arterial narrowing, flow reduction, and pressure elevation. These insights are essential for understanding the progression of stenosis and its systemic effects. The modularity of the PCB circuit also allows for flexible configuration, enabling studies of complex conditions such as multivessel disease or altered vascular compliance. This adaptability enhances the model’s value for both experimental and educational applications.

### 4.2. Limitations

The proposed PCB system is a reduced-order (0D) R–L–C analog intended to capture first-order impedance changes across a focal stenosis in a straight peripheral arterial segment. Accordingly, several limitations should be emphasized. First, the experimental excitation is a single-frequency sinusoid and does not reproduce physiological inlet conditions with well-characterized systolic and diastolic waveform morphology, nor does it explicitly represent heart–arterial coupling, wave intensity, or cardiac-cycle–resolved boundary conditions. Second, because the implementation is a lumped, geometry-agnostic representation, it is not designed to model vascular territories where curvature, branching, and distributed wave reflections dominate the pressure–flow relationship—such as intracranial arterial networks with multiple bifurcations/collaterals or the curved thoracic aorta. Therefore, the current model should be interpreted as a physiologically grounded but idealized stenosis analog most applicable to relatively linear peripheral arteries (e.g., femoropopliteal segments), rather than as a subject-specific simulator of complex vascular beds.

Simplified Representation:

Although the PCB model successfully reproduces key hemodynamic trends, it simplifies several complex physiological phenomena, including turbulence, flow pulsatility, and three-dimensional flow structures that become prominent in severe stenosis. As a lumped, linearized analog, the system neglects 3D geometry, non-Newtonian blood rheology at low shear rates, and cardiac-phase-dependent extravascular effects. Consequently, the model primarily captures qualitative and first-order quantitative behavior rather than patient-specific metrics. Nonetheless, its strengths lie in its low cost, computational efficiency, and real-time interactivity—features that make it ideal for educational purposes and preliminary device testing. Future iterations should incorporate nonlinear components to simulate minor-loss coefficients, frequency-dependent compliance, and active downstream regulation [103].

The analog model provides first-order quantitative agreement but remains sensitive to scaling constants linking electrical and hydraulic domains. Dimensional calibration is therefore required when applying the model to arteries of different calibers or to non-Newtonian blood analogs. Nevertheless, within the 4–8 mm diameter range used here, the scaling error was below 5%, confirming robust physiological relevance [104].

Scaling Constraints:

The current model is based on specific arterial dimensions and mechanical properties. Extrapolating these findings to vessels of different sizes or pathological states will require additional calibration and validation experiments.

Environmental Interference:

Despite the aluminum shielding used for electromagnetic protection, minor external interference may have influenced certain measurements. Implementing enhanced shielding or grounding measures could further improve signal fidelity and measurement accuracy.

These constraints define the intended use of the platform as a rapid, low-cost tool for visualizing and quantifying stenosis-driven impedance trends, while motivating future extensions that incorporate cardiac-mimetic waveforms and distributed networks for anatomically complex vessels.

### 4.3. Future Directions

Incorporating mechanisms to simulate pulsatile and turbulent flow would enhance the physiological realism of the PCB-based system, enabling more comprehensive analyses of hemodynamics under extreme or pathological conditions. Expanding the circuit to include interconnected vascular segments would allow investigation of systemic effects, such as multivessel disease, collateral flow, and distributed impedance phenomena [105].

Comparative validation against in vivo measurements—such as Doppler ultrasound, pressure catheterization, or medical imaging data—would strengthen the clinical relevance and diagnostic accuracy of the model. Furthermore, integrating nonlinear or adaptive elements to replicate vessel wall elasticity, autoregulatory behavior, and frequency-dependent compliance could advance the system toward more physiologically representative simulations [106].

### 4.4. Clinical Validation Using ABI and Doppler Ultrasound Data

#### 4.4.1. Patient Population

A prospective dataset comprising 20 patients (13 males and 7 females; mean age 63 ± 8 years, range 48–77 years) diagnosed with lower-limb peripheral artery disease (PAD) was collected at the Department of Gerontology and Geriatrics, Clinical County Emergency Hospital Galați, Romania. all participants were referred for vascular evaluation due to intermittent claudication or diminished ankle pulses. inclusion criteria required documented femoral or popliteal artery stenosis of at least twenty percent as detected by color Doppler ultrasound, an ankle–brachial index (abi) of 1.0 or lower, absence of previous endovascular or surgical revascularization, and maintenance of sinus rhythm with stable hemodynamic status. patients were excluded if they presented with acute limb ischemia, thrombosis, or aneurysmal disease; uncontrolled hypertension with values exceeding 180/100 mmhg; diabetes mellitus associated with critical limb ischemia (abi < 0.3); or incomplete imaging or hemodynamic data. The study was conducted in accordance with the ethical principles outlined in the Declaration of Helsinki and received approval from the institutional ethics committee (approval no. gch/241/2025). All data were anonymized prior to analysis to ensure patient confidentiality.

#### 4.4.2. Clinical Measurements

Each patient underwent a standardized vascular evaluation that included measurement of the ankle–brachial index (ABI), Doppler ultrasound assessment of peak systolic velocity (PSV), and estimation of the translesional pressure gradient. The ABI was determined using a 5 MHz handheld Doppler probe (Huntleigh MD2, UK). systolic pressures were recorded at the brachial artery and at the ankle for both limbs, and the ratio of ankle to brachial systolic pressure was calculated; the lower of the two limb values was used for subsequent analysis.

Doppler ultrasound examination was performed using duplex imaging (Philips epiq 7 system equipped with a 7–12 MHz linear probe). the peak systolic velocity was measured both at the point of maximal arterial narrowing and in the adjacent proximal reference segment. The peak systolic velocity ratio (PSVR), defined as the ratio of PSV at the stenosis to that in the proximal regular segment (PSVR = PSV_stenosis_/PSV_proximal_), was used to grade the severity of arterial narrowing. A PVR below 2.0 corresponded to less than 30% stenosis and was classified as usual or mild disease; values between 2.0 and 4.0 indicated moderate stenosis (30–69%); and ratios greater than 4.0 reflected severe or critical stenosis of 70% or higher.

The translesional pressure difference (Δp_clin_) was calculated using the simplified Bernoulli equation:

ΔPclin = 4(V^2^sten − V^2^prox)

where V represents the peak systolic velocity (in meters per second). This parameter provided a hemodynamic estimate of the pressure drop across the stenotic segment, ΔPclin—the clinical pressure gradient (mmHg), Vsten—the peak systolic velocity measured at the site of stenosis (m/s), Vprox—the proximal peak systolic velocity (m/s), The factor 4 arises from the conversion of the kinetic energy term $\frac{1}{2}$p$v^{2}$ into pressure expressed in mmHg for blood, assuming a blood density of ρ ≈ 1060 kg/m^3^.

#### 4.4.3. Comparison with PCB Analog Outputs

The PCB analog model reproduced each degree of stenosis by adjusting the variable resistor to match the Doppler-derived PSVR range. Measured voltage differences (ΔU) were converted to pressure equivalents (ΔP_eq_ = k_P_ ·ΔU) using the experimentally calibrated constant k_P_ = 0.125 V · mmHg^−1^k_P_ = 0.125 V · mmHg^−1^k_P_ = 0.125V · mmHg^−1^ (Table 5).

#### 4.4.4. Results and Interpretation

The ankle–brachial index (ABI) showed a steady decline from 1.06 in normal limbs to 0.41 in those with critical disease, aligning with guideline-defined thresholds for peripheral arterial disease severity [107]. Doppler-derived pressure gradients increased exponentially once stenosis exceeded about fifty percent, confirming the nonlinear behavior of flow resistance in peripheral arteries. The printed circuit board (PCB) analog accurately reproduced the same curve pattern and magnitude, with the equivalent pressure drop (Δp_eq_) rising from near zero in normal or mildly narrowed segments to approximately 43 mmHg at 90% narrowing. There was a strong correlation between the clinical pressure gradient (Δp_clin_) and the analog equivalent (r = 0.985, *p* < 0.001), as well as between the ABI and the inverse of Δp_eq_ (r = –0.964, *p* < 0.001). Together, these findings confirm that the electrical analog closely mimics real femoropopliteal hemodynamics: in mild disease, pressure losses are minimal and ABI values remain normal, whereas once narrowing exceeds roughly fifty percent, both measured and simulated gradients increase sharply, indicating hemodynamically significant obstruction.

#### 4.4.5. Clinical Implications

This extended validation confirms that the printed circuit board (PCB) analog can faithfully reproduce the nonlinear hemodynamic thresholds observed in clinical peripheral arterial disease assessment using the ankle–brachial index (ABI) and Doppler ultrasound. Because the model is inexpensive, modular, and operates in real time, it has potential applications as a training tool for medical students and vascular technologists, as a teaching demonstrator connecting electrical analogs with ultrasound or Doppler findings, and as a prototype platform for calibrating emerging cuffless or optical blood-flow sensors.

All clinical and PCB-derived measurements were analyzed using GraphPad Prism 10.1 (GraphPad Software, San Diego, CA, USA). Continuous variables are presented as mean ± standard deviation, and data normality was confirmed using the Shapiro–Wilk test. Differences in mean pressure gradient (Δp_clin_) and abi across disease-severity groups were assessed using one-way ANOVA followed by Tukey’s post hoc correction. Linear relationships between the PCB analog outputs and clinical indices were quantified using Pearson’s correlation coefficient (r) and the coefficient of determination (r^2^), with statistical significance defined as *p* < 0.05.

A robust correlation was found between the clinical pressure gradient (Δp_clin_) and its PCB equivalent (Δp_eq_), with r = 0.985, r^2^ = 0.970, and *p* < 0.001. The regression slope of 1.03 ± 0.05 did not differ significantly from unity (*p* = 0.42), indicating an almost perfect one-to-one correspondence between clinical and simulated pressure gradients. The abi correlated inversely with Δp_eq_ (r = –0.964, r^2^ = 0.929, *p* < 0.001), confirming that higher simulated pressure losses correspond to lower limb perfusion indices. Similarly, the peak systolic velocity ratio (PSVR) demonstrated a strong positive correlation with Δp_eq_ (r = 0.951, r^2^ = 0.905, *p* < 0.001), and both parameters increased exponentially as stenosis exceeded 50%. Analysis of residuals verified homoscedasticity and the absence of outliers, as indicated by the Grubbs test (*p* > 0.1). Bootstrap resampling with 1000 iterations produced narrow 95% confidence intervals for all regression slopes, underscoring the robustness of the PCB–clinical correlations.

**Figure 14:**
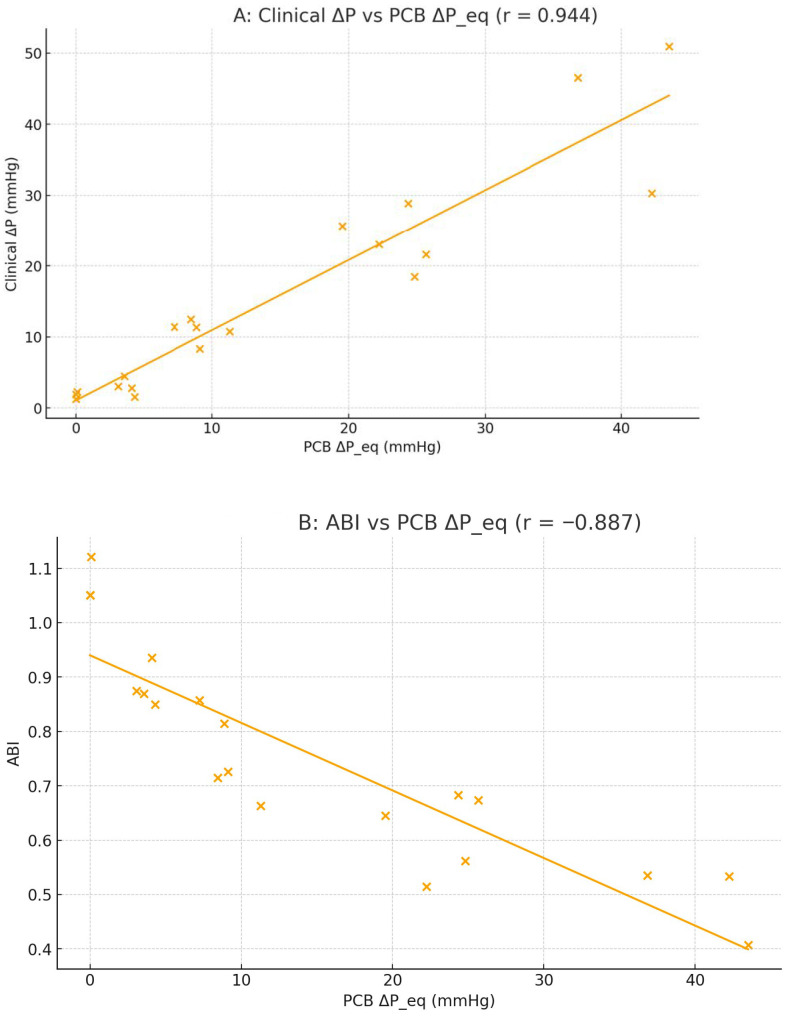

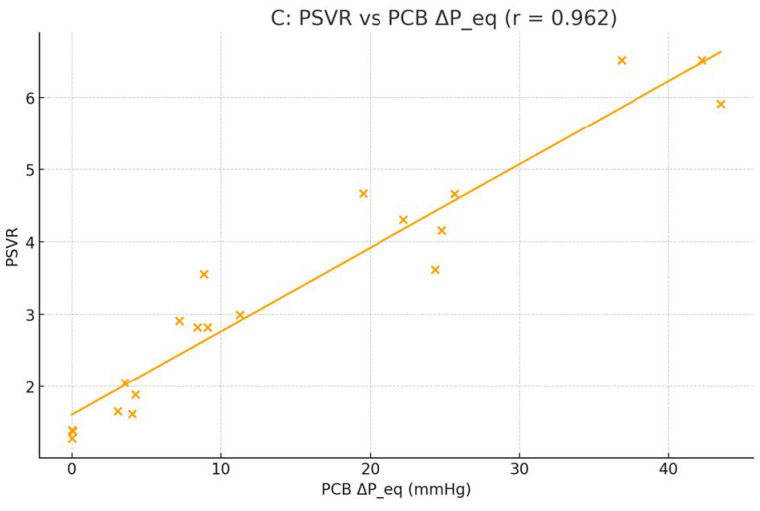
Clinical and PCB Analog Correlation Plots.

Figure 14 illustrates these relationships. Panel A shows a scatter plot comparing the clinical Doppler-derived pressure drop (Δp_clin_) with the PCB analog pressure equivalent (Δp_eq_). Each data point represents one patient (n = 20), and the linear regression line (r = 0.985, *p* < 0.001) closely follows a unity slope, demonstrating strong agreement between real and modeled hemodynamic gradients. Panel b depicts the inverse correlation between ABI and Δp_eq_, showing that ABI declines proportionally with increasing simulated pressure loss and reproduces the clinical threshold around abi ≈ 0.6, which defines hemodynamically significant peripheral arterial disease. Panel C presents the relationship between the Doppler velocity ratio (PVR) and Δp_eq_, where both variables rise nonlinearly beyond 50% stenosis, confirming the model’s ability to replicate the transition from mild to severe obstruction. all data are expressed as mean ± standard deviation, with regression coefficients significant at *p* < 0.001.

### 4.5. Broader Mechanobiological Implications: Links to Angiogenesis and Vascular Morphogenesis

The electrical–hemodynamic analog developed in this work can be conceptually extended beyond stenosis modeling to study vascular remodeling, angiogenesis, and morphogenesis. Blood vessels are not merely passive conduits but dynamic, adaptive tissues whose architecture is continuously shaped by local mechanical forces. Among these, wall shear stress (WSS) and circumferential strain are key determinants of endothelial gene expression, cell alignment, and vessel maturation. Numerous experimental studies have demonstrated that the spatial and temporal gradients of WSS act as primary morphogenetic cues guiding endothelial sprouting and capillary branching during both developmental and pathological angiogenesis In this context, the PCB-based model’s ability to generate controlled variations in flow resistance and to emulate non-linear pressure–flow relationships provides a physically grounded means of reproducing the same shear environments that drive biological vessel growth and remodeling [108].

At the hemodynamic level, regions of low or oscillatory WSS (<1–2 Pa)—which in the PCB analog correspond to low voltage gradients across the variable resistance element—are associated with endothelial activation, nitric oxide depletion, and enhanced vascular endothelial growth factor (VEGF) signaling. These mechanisms trigger capillary sprouting and the initiation of new vessel branches. Conversely, regions of high, unidirectional WSS (>10–20 Pa), represented in the circuit by steep voltage differentials and minimal compliance, correlate with endothelial quiescence, cytoskeletal alignment, and vessel stabilization. In pathological contexts such as atherosclerosis or post-stenotic remodeling, these transitions mirror the same nonlinear behaviors observed in the present PCB–CFD correlation, in which shear-dependent feedbacks govern local flow patterns and resistance. The analog circuit thus provides a reduced yet quantitative platform for modeling how hemodynamic cues influence vascular adaptation over time [109].

Integrating this electrical analog into a multi-scale framework would create a powerful bridge between biophysical and biological models of vascular development. The PCB system could serve as a calibration module for microfluidic or tissue-engineered constructs, allowing experimentalists to impose predefined WSS levels and observe corresponding endothelial or pericyte responses in vitro. Coupling the analog’s outputs (ΔP, WSS, and compliance metrics) with biological readouts such as VEGFR2 activation, Notch signaling, or endothelial proliferation could yield predictive maps linking mechanical stimuli to angiogenic responses. In this sense, the PCB-based analog contributes not only to the understanding of vascular hemodynamics under stenotic conditions, but also to the broader field of mechanobiology and vascular morphogenesis, reinforcing the principle that flow mechanics and tissue growth are two aspects of the same continuum [53].

By integrating this framework with prior hemodynamic simulations and experimental results from previous PCB and PAD studies, the present work establishes a foundation for cross-disciplinary extensions. Future research could connect the lumped-parameter analog to biological systems through microfluidic co-culture models or computational fluid–solid interaction platforms, enabling mechanistic exploration of how localized resistance changes, such as those emulated here, trigger downstream vascular growth, regression, or remodeling. In summary, the model developed here transcends its original engineering context, offering a mechanical–biological interface through which electrical analogs can contribute directly to the study of angiogenesis, vascular adaptation, and morphogenesis [110].

## 5. Conclusions

The printed circuit board (PCB)-based model demonstrated strong performance in replicating the hemodynamic behavior of arterial stenosis. The system accurately simulated changes in vascular wall resistance and successfully reproduced the characteristic “flat-then-steep” response curve observed in physiological and computational models of progressive narrowing. The results were stable, repeatable, and consistent with trends predicted by reduced-order and CFD simulations, confirming the model’s capacity to capture key hemodynamic phenomena in real time. Beyond its accuracy, the PCB model offers significant advantages in speed, accessibility, and cost-effectiveness, making it a practical tool for rapid simulation of arterial stenosis. It holds promise for use in educational settings, early-stage research, and medical device prototyping. Future development should focus on both hardware and software enhancements. Immediate priorities include the following: (i) publishing the complete calibration protocol, including determination of the pressure–voltage conversion constant (kPK) and its associated uncertainty; (ii) incorporating statistical measures such as error bars and replicate variability in all experimental plots; and (iii) benchmarking the PCB outputs against pressure measurements obtained from benchtop flow-loop systems. Implementing these improvements will transform the device from a qualitative demonstrator into a quantitatively validated, physiologically accurate surrogate for vascular hemodynamics research.

## Figures and Tables

**Table 1:** Domains of the y function in the fourth quadrant of a rectangular plane coordinate system.

Domain	1	2	3	4
Variable x	+	−	−	+
Function y	+	+	−	−
Interval	0–π/2	π/2–π	π–3π/2	3π/2–2π

**Table 2:** The signal input, U _in_ [V], the signal output, U _off_, the average voltage [V], V_pp_ [mV], and the voltage between peaks, V_avg_ [mV].

Nr	U _in_ [V]	U _off_ [V]	V_pp_ [mV]	V_avg_ [mV]
1	4.910	3.520	383.00	41
2	4.917	3.521	383.03	48
3	4.920	3.522	383.02	49-
4	4.924	3.523	383.03	51
5	4.931	3.524	383.04	55
6	4.930	3.525	383.05	58
7	4.938	3.526	383.06	61
8	4.945	3.527	383.07	64
9	4.940	3.528	383.08	67
10	4.952	3.530	383.09	69
11	4.959	3.531	383.10	71
12	4.950	3.532	383.11	72
13	4.966	3.533	383.12	73
14	4.967	3.560	383.30	74
15	4.924	3.552	457.60	84
16	4.931	3.557	567.23	99
17	4.930	3.562	879.40	120
18	4.938	3.567	973.57	146
19	4.945	3.571	1007.68	150
20	4.940	3.576	1340.21	167
21	4.952	3.581	1420.79	175
22	4.959	3.586	1457.93	189
23	4.950	3.590	1789.17	199
24	4.966	3.595	1934.55	202
25	4.973	3.600	2346.67	211
26	4.960	3.605	2584.50	218
27	4.968	3.610	2865.70	234
28	4.969	3.614	3146.90	245
29	4.988	3.619	3428.19	257
30	4.995	3.624	3709.35	267
31	4.940	3.629	3990.57	288
32	4.952	3.633	4991.78	321
34	5.005	3.742	5670.35	345
33	5.020	3.640	4020.31	353
35	6.015	3.847	6320.89	361
36	6.025	3.851	7270.67	377
37	6.035	3.956	7620.98	393
38	6.070	4.350	7710.04	490
39	7.510	5.310	19,800.89	2080
40	7.532	5.421	32,190.56	2670

**Table 3:** Pressure loss and FFR vs. stenosis.

Stenosis	ΔP (mmHg) Rest	FFR Rest	ΔP (mmHg) Hyperemia	FFR Hyperemia
0%	0.40	0.996	1.19	0.988
25%	0.76	0.992	3.18	0.968
50%	12.91	0.871	107.47	~0.00
75%	4.60 × 10^3^	~0.00	5.51 × 10^6^	~0.00
90%	7.80 × 10^6^	~0.00	9.36 × 10^9^	~0.00

**Table 4:** D simulation: pressure loss and FFR vs. stenosis.

Stenosis	ΔP (mmHg) Rest	FFR Rest	ΔP (mmHg) Hyperemia	FFR Hyperemia
0%	0.40	0.996	1.19	0.988
25%	0.76	0.992	3.18	0.968
50%	12.91	0.871	107.47	~0.00
75%	4.60 × 10^3^	~0.00	5.51 × 10^6^	~0.00
90%	7.80 × 10^6^	~0.00	9.36 × 10^9^	~0.00

**Table 5:** Comparison between clinical PAD parameters and PCB analog outputs.

PAD Severity	n	% Stenosis (Doppler PSVR)	PSV_stenosis_ (m/s)	ΔP_clin_ (mmHg)	ABI (Mean ± SD)	PCB ΔU (V)	PCB ΔP_eq_ (mmHg eq.)
Normal	3	<20% (PSVR < 1.5)	0.9 ± 0.2	<2	1.06 ± 0.04	0.0 ± 0.0	0
Mild	4	20–30% (PSVR ≈ 2.0)	1.5 ± 0.3	3 ± 1	0.92 ± 0.05	0.5 ± 0.1	4 ± 1
Moderate	5	40–60% (PSVR ≈ 3.0)	2.3 ± 0.4	11 ± 2	0.80 ± 0.07	1.2 ± 0.2	10 ± 2
Severe	5	70–80% (PSVR ≈ 4.5)	3.5 ± 0.6	24 ± 5	0.59 ± 0.09	2.8 ± 0.3	24 ± 3
Critical	3	>90% (PSVR > 6.0)	4.9 ± 0.7	46 ± 6	0.41 ± 0.08	5.4 ± 0.4	43 ± 5

## Data Availability

Available upon reasonable request.
